# AI-driven rational design of promiscuous and selective plastic-binding peptides

**DOI:** 10.1039/d5sc04903b

**Published:** 2025-10-01

**Authors:** Vinamr Jain, Michael T. Bergman, Carol K. Hall, Fengqi You

**Affiliations:** a College of Engineering, Cornell University Ithaca New York 14853 USA fengqi.you@cornell.edu; b Department of Chemical and Biomolecular Engineering, North Carolina State University Raleigh NC 27606 USA; c Robert Frederick Smith School of Chemical and Biomolecular Engineering, Cornell University Ithaca New York 14853 USA; d Cornell University AI for Science Institute, Cornell University Ithaca New York 14853 USA; e Cornell AI for Sustainability Initiative (CAISI), Cornell University Ithaca New York 14853 USA

## Abstract

Microplastic pollution is challenging to remediate due to the small size and heterogeneous composition of microplastic particles. Remediation efforts would benefit from tools that either bind to the many components of microplastic pollution (promiscuous binding) to facilitate quantitation and capture, or bind to certain components of pollution (selective binding) to facilitate separation or degradation. Such a role could be filled by polypeptides, which can bind selectively or promiscuously to biomolecules or materials. While methods exist to design plastic-binding peptides (PBPs) for a single plastic, the design of promiscuous plastic-binding peptides has received scant attention, and there are no methods to design selective plastic-binding peptides. Here, we present a minimalist yet high-performing framework integrating Long Short-Term Memory (LSTM) models with simulated annealing (SA) to design promiscuous plastic-binding or selective plastic-binding peptides. Our approach learns sequence–function relationships governing peptide affinity for different plastics from PepBD data, a biophysical modeling program. The learned relationship enables rapid design of peptides with tailored binding properties for arbitrary combinations of plastics. We use our LSTM-SA framework to engineer (1) promiscuous plastic-binding peptides with affinity for five plastics (polyethylene, polypropylene, PET, polyvinyl chloride, and nylon), and (2) selective plastic-binding peptides that bind preferentially to one plastic (polypropylene) over another (PET). Notably, the promiscuous plastic-binding peptides are the first reported designs intended to bind to nylon and PVC. Molecular dynamics simulations validate that our designed peptides exhibit the predicted binding behaviors, where high affinity is linked to strong van der Waals interactions. The framework's modularity can be readily adapted to optimize peptide selectivity or promiscuity for different combinations of plastics. More broadly, the architecture may be useful for designing peptides that bind to other solid materials.

## Abstract 2

An AI-driven LSTM-SA framework rapidly designs peptides with either broad or selective binding to plastics, offering a versatile strategy for microplastic capture and separation.

## Graphical abstract

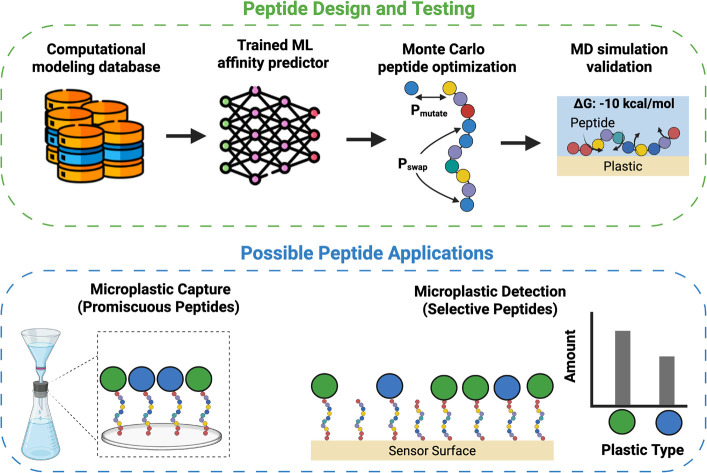

## Introduction

Microplastic contamination threatens biodiversity, food safety, human health, and marine environments.^1^ Synthetic polymers such as polyethylene terephthalate, polyethylene, and polypropylene pervade aquatic ecosystems.^2–4^ Microplastics can induce oxidative stress and cytotoxicity in marine organisms,^5^ affecting their reproductive health and survival.^6^ Microplastic ingestion by marine species may lead to the transfer of microplastics through the food chain, potentially leading humans to consume microplastics.^7^ Strategies to remediate microplastic pollution must be developed to avoid potential harm to the environment and human health.^8–10^

Plastic-binding peptides (PBPs) could be useful for remediating microplastic pollution.^11^ Polypeptides readily adsorb to micro- and nanometer sized materials,^12^ suggesting that they could help detect, capture, and/or biodegrade microplastic pollution. For example, plastic-binding peptides have been used to help detect microplastic pollution^9,10^ and accelerate enzymatic degradation of the plastic PET.^11^ They could also augment protein-based strategies for water purification.^12,13^ Polypeptides are biocompatible, so they themselves will not negatively impact the environment, could be engineered into the genome of microorganisms being used to combat microplastic pollution,^13–15^ and may detect or capture microplastics in biological systems. There is also flexibility in manufacturing and applying PBPs, since peptides can be synthesized either chemically or biologically.

Before applying PBPs to microplastic remediation, they must first be discovered. Solid-binding peptides are typically found *via* high-throughput screening (HTS) methods like phage-display.^16^ Although HTS has found peptides that bind to many materials,^17^ it has limitations. HTS may not discover many high-affinity solid-binding peptides since it randomly samples a small fraction of the 20^N^ possible N-residue peptides. HTS provides little to no insight into the basis of peptide affinity for a material, meaning it must be repeated if peptides with properties like binding selectivity or promiscuity are desired. Computational tools are an appealing alternative to HTS for discovering PBPs because they can intelligently explore peptide sequence space and provide insight into the physical basis of peptide affinity. Many computational tools—molecular docking, molecular dynamics (MD) simulations, machine learning (ML), evolutionary algorithms, and generative models—have been applied to design and evaluate peptides targeting proteins, small molecules, nanoparticles, and other materials.^18–24^ However, computational methods face their own challenges when designing PBPs, the most notable of which is the scarcity of experimental data. There is a small amount of HTS data for PBPs that bind to polypropylene and polystyrene,^25–28^ but there is no data for other common plastics. Without such data, machine learning tools that have revolutionized protein design^29,30^ and drug design^31,32^ cannot be transferred to PBP design.

Recent work has shown how PBPs and other solid-binding peptides can be designed with computational methods. One approach is to train ML models on HTS data, which has led to ML-based classifiers that can predict if a peptide binds to polystyrene^33,34^ or gold^35^ and an ML model that generated novel quartz-binding peptides based on sequence patterns in HTS data.^36^ A second approach is to collect a small experimental dataset of quantitative affinity measurements that subsequently guides sampling of peptides. Examples include Bayesian optimization of peptide selectivity between gold and silver surfaces,^37^ and the design of iron-oxide binding peptides.^38^ A third approach is to apply biophysical modeling that pairs Monte Carlo sampling of peptide sequences and conformations with molecular mechanics force fields to search for peptides with strong binding energies to a given material. Examples include PepBD^39^ or RosettaSurface.^40^ A fourth approach, which we take in this work, is to use biophysical modeling to generate a dataset that trains ML models that search for PBPs. For example, Conchello Vendrell *et al.* developed a hybrid variational autoencoder plus quantum circuit model which was trained on PepBD data to identify PET-binding peptides,^41^ Alshehri *et al.*^42^ trained an evidential deep learning model on PepBD data to discover PBPs with 5–34% stronger affinities than the best PepBD designs for several plastics, and Dhoriyani *et al.* combined biophysical Potts models with quantum annealing and reinforcement learning to discover plastic-binding peptides.^43^

Computational PBP discovery has, however, neglected two classes of PBPs that could greatly aid microplastic remediation efforts. The first class is PBPs that bind to multiple types of plastic, which we term “promiscuous plastic-binding peptides”. As microplastic waste is typically composed of several types of plastic,^44,45^ promiscuous plastic-binding peptides could more comprehensively address microplastic waste compared to single-plastic binding peptides. Motivated by the recent design of PBPs with high affinity for polyethylene and polypropylene,^46^ we aim in this work to design PBPs that bind to five types of plastic. The second class of PBPs is the converse of the first class: peptides that bind preferentially to one plastic over others, or “selective plastic-binding peptides”. Such peptides could help separate microplastic waste into plastic components,^47^ or help plastic-degrading enzymes^48^ and microorganisms^15^ adhere to the particular plastic that they degrade. We expect that there are promiscuous or selective peptides since polypeptides can discriminate between plastics or other materials.^49–51^

The goal of the present work is to design promiscuous- and selective plastic-binding peptides. Such peptides cannot be developed with existing tools. Extending biophysical modeling methods to design such peptides may be challenging. Quantifying selectivity or promiscuity requires evaluating peptide affinity for multiple plastics, which likely requires time-consuming sampling of peptide conformational space since a peptide may adopt different adsorbed conformations to different plastics. Past work has suggested that this issue could be circumvented by altering the optimization function,^52,53^ but we show here that ML offers a simple and appealing solution. Meanwhile, previous ML methods for PBP design were only intended to optimize affinity for a single plastic. Generally, multi-objective optimization of solid-binding peptides within ML remains relatively unexplored, with the exception of a few studies.^37^

In this work, we integrate a Long Short-Term Memory (LSTM) model with simulated annealing (SA) to design promiscuous- and selective plastic-binding peptides. We train LSTM models on PepBD biophysical modeling data to predict peptide affinity scores based on the peptide sequence. The learned sequence:function relationship guides SA to maximize either the average affinity for multiple plastics (promiscuous plastic-binding peptides) or the affinity difference between two plastics (selective plastic-binding peptides). We apply this framework to design promiscuous plastic-binding peptides that bind to polyethylene (PE), polypropylene (PP), polyethylene terephthalate (PET), polyvinyl chloride (PVC), and nylon 6-6 (nylon), representing the first study to report peptide affinity to nylon and PVC. We also design selective plastic-binding peptides that bind with higher affinity to PP than to PET. The promiscuity or selectivity of the PBPs designed by the LSTM-SA model are validated in MD simulations. Subsequent analysis sheds light on the basis for selectivity or promiscuity by revealing the role of amino acid composition and van der Waals interactions in determining binding behavior. Overall, the low complexity, speed, and modularity of the LSTM-SA model compares favorably to the complex ML architectures designed for other peptide design tasks.^54^ A complex ML model is not necessary for effective PBP design. While the focus lies solely only on PBPs in this work, the LSTM-SA model may be extended to design other solid-binding peptides, which have many uses in biomaterials and biotechnologies.^16,17^

## Results and discussion

### An LSTM-SA framework for generating promiscuous plastic-binding and selective plastic-binding peptides

**Fig. 1:**
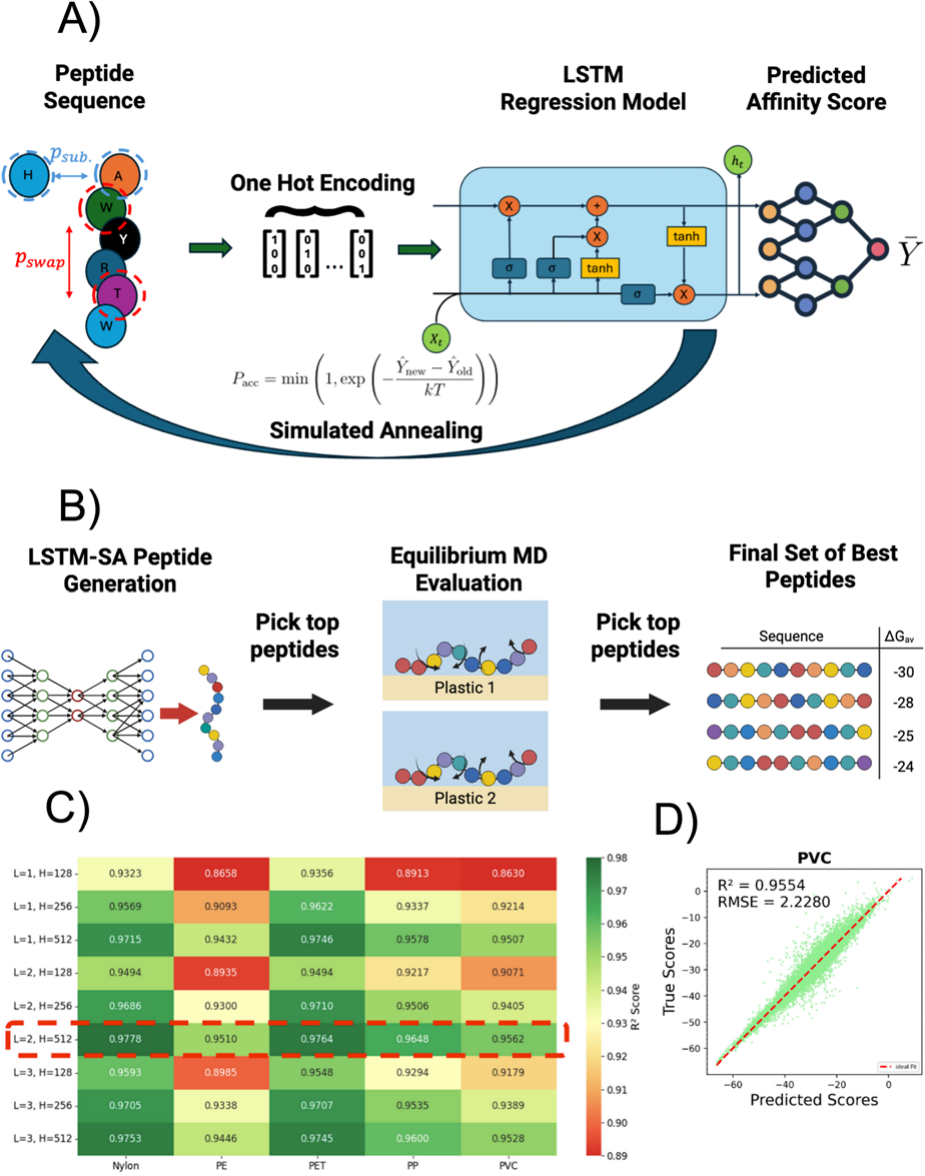
Overview of the LSTM-SA framework for generating plastic-binding peptides. (A) LSTM-SA model architecture. Peptides are encoded as one-hot vectors and input into a Long Short-Term Memory (LSTM) regression model that predicts the PepBD affinity score. The LSTM is trained on PepBD data, then serves as a surrogate model for affinity score prediction during peptide design. Peptides are generated using simulated annealing (SA), which begins with a random amino sequence then iteratively swaps or replaces amino acids. Changes are accepted or rejected based on the Metropolis criterion to maximize binding promiscuity or selectivity. (B) Peptide design-validate procedure. The best peptides generated by the LSTM-SA model undergo MD simulations to evaluate their affinity to all target plastics. Peptides with the best simulation results may undergo further computational or experimental validation. (C) *R*^2^ values over the validation dataset during a hyperparameter grid search of LSTM's number of layers (1 to 3) and hidden dimension (128 to 512). As indicated by the dashed red box, LSTM with 2 layers and hidden dimension 512 predicts the affinity score accurately for all plastics. (D) Scatterplot of affinity score predictions *versus* actual scores over the test set using LSTM for the plastic PVC. The predicted scores almost perfectly align with the true scores, with an RMSE of 2.2. Performance is similar for the other plastics (Fig. S4).

We present an LSTM-SA model that designs peptides with promiscuous or selective binding to plastics. Our computational framework combines Long Short-Term Memory (LSTM) neural networks^55^ that predict peptide affinity to plastics based on the amino acid sequence, and simulated annealing^56^ (SA) to generate peptides predicted to have promiscuous- or selective binding to plastics (Fig. 1A). We summarize key features of the framework here, and details can be found in SI Methods. The LSTM is trained on PepBD data (whose physicochemical properties are analyzed in Fig. S1 and S2) to predict the PepBD affinity score for a given plastic using a one-hot encoding of the peptide's amino acid sequence. A separate LSTM is trained for each plastic, and example training and validation loss curves are shown in Fig. S3. The PepBD affinity score^57^ is a sum of the peptide adsorption enthalpy and internal peptide energy, where both terms are comprised of coulombic, Lennard-Jones, and generalized Born solvent energies. The lower the value of the PepBD affinity score, the higher the predicted affinity for a plastic. Peptides are generated by using SA to find amino acid sequences with one of two metrics: a high average affinity score to a set of plastics (promiscuous plastic-binding peptides), or a large affinity score difference between two plastics (selective plastic-binding peptides). SA starts with a random amino acid sequence, randomly mutates the sequence, and then accepts or rejects the mutation based on the Metropolis criterion and the change in the metric. Calculation of the metric uses the trained LSTM score predictors for all plastics considered during design. Each SA run attempts 21 525 sequence mutations and takes 25 848 seconds (∼7.2 hours) to design promiscuous plastic-binding, and 3837 seconds (∼1.1 hours) to design selective plastic-binding peptides (Table 1). Fifty-five runs were performed for each design goal. Of the top 1000 best-scoring designed promiscuous or selective plastic-binding peptides, none were found in the PepBD dataset, indicating the novelty of the generated peptides. All designed peptides contain 12 residues, matching the length of the peptides in the PepBD datasets.

**Table 1:** Performance evaluation of the LSTM-SA model

Plastic	Total runtime (s)	Novelty	Score: mean ± std (minimum)	
			Top 100 PepBD	Top 100 generated
PET	11 191.76	0.990	−61.2 ± 1.3 (−63.8)	−62.0 ± 0.4 (−64.1)
PE	11 189.97	0.987	−55.7 ± 1.5 (−59.6)	−58.34 ± 0.5 (−60.5)
PP	11 199.70	0.995	−48.1 ± 1.4 (−52.3)	−50.7 ± 0.3 (−52.2)
PVC	11 197.80	0.998	−61.2 ± 2.3 (−66.3)	−66.0 ± 0.2 (−66.9)
Nylon	11 194.92	0.986	−69.6 ± 1.8 (−74.3)	−73.1 ± 0.3 (−74.1)
Multi	25 848.28	1.00	N/A^a^	−50.3 ± 0.6 (−51.8)
PP–PET	3837.32	1.00	N/A^a^	−36.7 ± 5.6 (−46.0)

a PepBD has not designed such peptides.

Designed PBPs are validated in MD simulations by calculating their adsorption free energies (Δ*G*) using the MM/GBSA method^58^ over an ensemble of simulations (Fig. 1B). The computational cost of MD simulations and the need to simulate each peptide with multiple plastics means that only a small sample size can be evaluated. We evaluated 12 peptides for each design goal. Peptides were selected for evaluation by iteratively selecting the best-scoring peptide that differed by at least 3 amino acids from already selected peptides, thus giving a diverse set of peptides. The peptides with either the best average Δ*G* (promiscuous plastic-binding peptides) or largest Δ*G* difference (selective plastic-binding peptides) in MD simulations are identified as the best designs. We further evaluated three selective plastic-binding peptides using steered MD^59^ (SMD) simulations to more rigorously calculate Δ*G*.

Two key features distinguish the LSTM-SA framework from biophysical modeling approaches. First, the LSTM predicts peptide affinity scores using only the amino acid sequence. The LSTM aggregates PepBD data from many adsorbed peptide conformations, implicitly learning how to predict peptide affinity to plastic using only the amino acid sequence and not the peptide's adsorbed conformation. Removing modeling of the peptide conformation accelerates peptide screening and makes it simple to calculate peptide affinity to multiple plastics simultaneously. Second, we train a separate LSTM to predict affinity scores for each plastic, so the different LSTMs can be used in a plug and play fashion. The combination of the two features makes it straightforward to design promiscuous and selective peptides. Designing such peptides using biophysical modeling would face the challenge of determining the stable adsorbed conformations of a peptide on multiple plastics, since a peptide will likely adopt a different stable adsorbed conformation (or conformations) on different plastics. The purpose of the LSTM-SA framework is not to generate peptides with higher affinity for single plastics than PepBD, but to optimize peptide affinity for multiple plastics.

### LSTM hyperparameter optimization and superior performance of LSTM over other ML models

We validate the efficacy of the LSTM to predict PepBD affinity scores across five plastics: PET, PE, PP, PVC, and nylon. PepBD data for PE, PET, and PP were taken from previous work,^39^ while data for PVC and nylon were generated for this work (see SI for details). We optimized the LSTM hyperparameters by conducting a grid search over the number of layers (1–3) and hidden dimensions (128–512). Performance of each LSTM architecture was evaluated using the coefficient of determination (*R*^2^) value between the predicted and true affinity scores in a validation data set (Fig. 1C). Performance is sensitive to both the hyperparameters and the plastic. The architecture with 2 layers and a hidden dimension of 512 (dashed red box in Fig. 1C) appears optimal: evaluation over multiple test datasets gives an *R*^2^ value greater than 0.95 for all plastics and a root mean square error (RMSE) of 2 relative to true PepBD affinity scores (Fig. 1D). This LSTM architecture is used in the remainder of the work.

We compared the performance of the LSTM to other recurrent neural networks (RNNs): the Bidirectional LSTM (BiLSTM), Gated Recurrent Unit (GRU), standard RNN, and Transformer (Table 2). All models use the optimal hyperparameters (2 layers and a hidden dimension of 512). Model performance was quantified *via* training time, *R*^2^ values, and the RMSE of affinity score predictions to true scores across all plastics. The LSTM consistently outperformed all alternatives: it had the highest *R*^2^ values (0.952 to 0.977) and the lowest RMSE values (1.8–2.2) across all plastics, and it only required a moderate training time. BiLSTM and GRU models showed slightly inferior performance to the LSTM, while the standard RNNs and Transformer architectures performed much worse. The RNN may have struggled to capture long-range dependencies in peptide sequences, while the Transformer may not have had sufficient training data to fit its large parameter space (Table S1), a known challenge for models with limited inductive biases.^60,61^ Alternatively, its self-attention mechanism may be suboptimal for learning the sequence:score relationship, especially when compared to the inherent sequential processing capabilities of LSTMs.^62^

**Table 2:** Performance of different RNN architectures across plastic datasets

Plastic	Dataset size (train/val/test)	Model	Training time (s)	*R* ^2^	RMSE
PET	353 581/44 198/44 198	LSTM	7474	**0.9755**	**2.23**
		BiLSTM	12 037	0.9723	2.37
		GRU	6586	0.9706	2.45
		RNN	1713	0.0802	13.69
		Transformer	8782	0.1274	13.34
PE	572 406/71 551/71 551	LSTM	25 947	**0.9517**	**2.24**
		BiLSTM	15 951	0.9382	2.53
		GRU	19 317	0.9415	2.46
		RNN	2051	0.5438	6.87
		Transformer	7268	0.4850	7.30
PP	346 789/43 349/43 349	LSTM	17 000	**0.9640**	**1.94**
		BiLSTM	10 697	0.9550	2.18
		GRU	9860	0.9541	2.20
		RNN	1031	0.5431	6.94
		Transformer	9312	0.3845	8.05
PVC	166 886/20 861/20 861	LSTM	10 253	**0.9554**	**2.23**
		BiLSTM	5552	0.9499	2.36
		GRU	3113	0.9502	2.35
		RNN	646	0.0000	10.55
		Transformer	1829	0.2226	9.30
Nylon	114 091/14 261/14 261	LSTM	6171	**0.9774**	**1.79**
		BiLSTM	3738	0.9714	2.01
		GRU	2309	0.9710	2.03
		RNN	359	0.6779	6.76
		Transformer	810	0.0000	11.91

### Design of promiscuous plastic-binding peptides

Our first goal was to design promiscuous plastic-binding peptides (or promiscuous peptides, for short). We envision that such peptides could be useful for remediating microplastic pollution. Such waste is often composed of multiple types of plastic, so a peptide that binds to all or many components would simplify remediation efforts. Our particular goal was to design peptides that bind to five common plastics: PE, PP, PET, PVC, and nylon. We designed such peptides by using the LSTM-SA framework to find peptides with a high average affinity score to the five plastics.

**Fig. 2:**
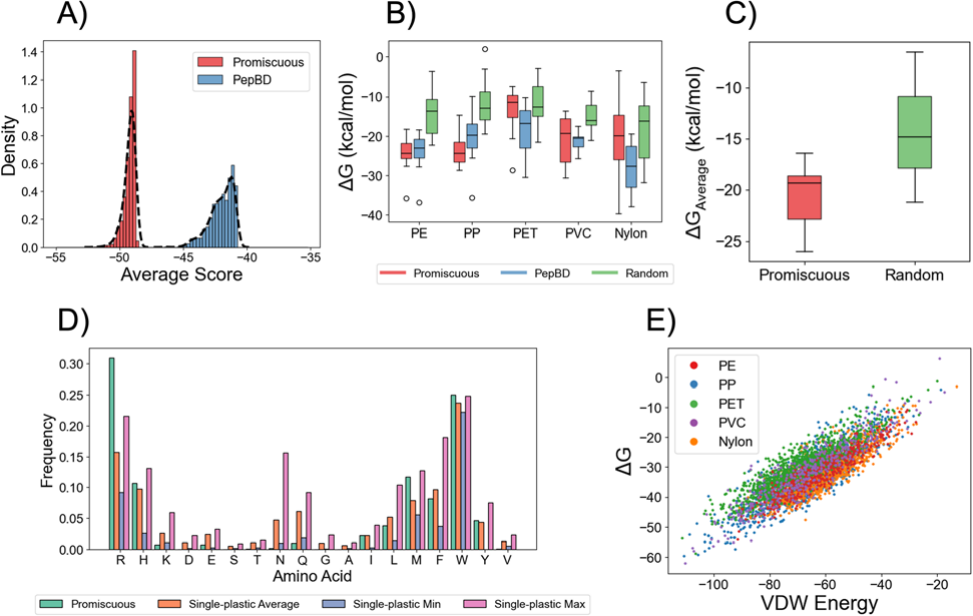
Design and validation of promiscuous plastic-binding peptides. (A) Comparison of average affinity scores over the five plastics predicted by the trained LSTMs. Two peptide categories are analyzed, each containing 1000 peptides: PepBD and promiscuous peptides. (B) MM/GBSA adsorption free energies (Δ*G*) for random, PepBD, and promiscuous peptides to PE, PP, PET, PVC, and nylon. The PepBD peptides differ between plastics, while random and promiscuous peptides were the same for all plastics. Twelve peptides in each category were evaluated. (C) Average Δ*G* over the five plastics for promiscuous and random peptides. PepBD data is not provided as the PepBD peptides in panel (B) differed for each plastic. (D) Amino acid composition of the 100 best promiscuous peptides, and comparison to the average, maximum, and minimum amino acid frequencies for the single plastic-binding peptides. (E) Correlation between the van der Waals (VDW) intermolecular energy and Δ*G* over all MD simulations for random, promiscuous, and PepBD peptides for each plastic.

The promiscuous peptides designed by the LSTM-SA model are predicted to have good affinity for four of the five plastics. To provide reference for the average affinity scores to five plastics, we predicted the average affinities of every peptide in the PepBD dataset (∼2.3 million) using the trained LSTM models, then selected the 1000 peptides with the best average affinity score. The designed promiscuous peptides have better (*i.e.*, more negative) average affinity scores than PepBD peptides (Fig. 2A). Comparing scores for individual plastic shows that the promiscuous peptides have better predicted affinity than PepBD peptides for all plastics but PET (Fig. S5A). To investigate if the LSTM-SA model generally struggled to design peptides for PET, we generated a new batch of “single plastic-binding peptides” for PET, *i.e.*, only affinity for PET was optimized during SA. The high predicted scores of these designs (Fig. S6A) indicates that the promiscuous peptides have poor affinity for PET due to the multi-objective aim of high affinity to five plastics, not a shortcoming of the LSTM-SA model. A second impact of multi-objective optimization is that the promiscuous peptides have notably lower affinity scores to each plastic than the best PepBD designs for the same plastic (Fig. S5B). It appears that optimizing affinity for multiple plastics reduces affinity to each individual plastic.

The promiscuous peptides show high affinity to three of the five plastics in MD simulations. Fig. 2B compares Δ*G* of the promiscuous peptides, random amino acid sequences (random), and the best-scoring PepBD peptides for each plastic. The data for random and PepBD peptides for PE, PP, and PET were taken from previous work.^42^ For PE, PP, and PVC, the promiscuous peptides have equal or better Δ*G* than PepBD peptides and consistently have better Δ*G* than random peptides. A conclusion cannot be made for nylon due to the large variability in Δ*G*. For PET, the promiscuous peptides have less favorable Δ*G* than PepBD peptides and comparable Δ*G* as the random peptides, in agreement with the poor affinity scores of the designs for PET (Fig. S5). The low affinity of the promiscuous peptides for PET does not mean the LSTM-SA model is incapable of generating peptides with high affinity for PET, since the single plastic-binding peptides for PET (see previous paragraph) have affinity comparable to the best PepBD designs (Fig. S6B and S2). The low affinity that the promiscuous peptides have for PET instead seems to arise from the difficulty of finding a peptide with high affinity for several plastics, akin to Fig. 2B. In total, the promiscuous peptides show high affinity for the plastics (except PET and possibly nylon). This is demonstrated by comparing the average Δ*G* over the five plastics for promiscuous and random peptides (Fig. 2C). Three exemplar promiscuous plastic-binding peptides are listed in Tables 3 and S2 compares Δ*G* of the promiscuous and random peptides for each plastic.

**Table 3:** Promiscuous plastic-binding peptides with high affinity for multiple plastics^a^

Sequence/description	Δ*G* PE ± 4.1 kcal mol^−1^	Δ*G* PP ± 4.3 kcal mol^−1^	Δ*G* PET ± 4.8 kcal mol^−1^	Δ*G* PVC ± 5.1 kcal mol^−1^	Δ*G* nylon ± 5.7 kcal mol^−1^	Δ*G* average kcal mol^−1^
Average random^b^	−14.6	−11.6	−11.9	−15.2	−18.7	−14.4
Best PepBD^c^	−36.8	−35.6	−30.4	−25.7	−37.9	—
Average PepBD^d^	−23.5	−20.3	−17.9	−21.3	−28.0	—
YWYERIFWRRMW	−35.8	−20.7	−20.6	−30.5	−22.4	−26.0
WRWHRMMHLRMW	−24.3	−25.3	−7.5	−26.9	−39.7	−24.7
RHRWLHWFLRMW	−27.7	−22.5	−28.6	−19.4	−24.8	−24.6

a Uncertainty in Δ*G* calculated from the mean average error between two evaluations of the 12 random peptides performed separately for each plastic.

b Average Δ*G* out of all random peptides in Fig. 2B.

c Lowest Δ*G* out of all PepBD peptides in Fig. 2B.

d Average Δ*G* out of all PepBD peptides in Fig. 2B.

The promiscuous peptides favor amino acids that are consistently found in the best-scoring peptides designed for each individual plastic, and their high affinity for the plastics stems from strong van der Waals interactions. Comparing the amino acid compositions of promiscuous peptides to that of the peptides generated by the LSTM-SA model when optimizing affinity for a single plastic (“single plastic-binding peptides”) reveals two notable properties (Fig. 2D). First, amino acids that frequently appear in single plastic-binding peptides for all plastics also appear frequently in promiscuous peptides, such as such as arginine (R), methionine (M), and tryptophan (W). This correlation is strong (*r*^2^ = 0.91, *p* < 10–6). SHAP analysis proposes that W and R are the dominant contributors to binding affinity, and suggests that some amino acid positions in the peptide sequence have more importance than others for plastic binding (Fig. S7). Second, amino acids that appear with disparate frequencies in single-plastic designs for different plastics do not appear frequently in promiscuous peptides. Avoiding such amino acids likely prevents a peptide from weakly interacting with one of the target plastics. Examples of this trend include asparagine (N) and glutamine (Q), although there are counterexamples like histidine (H) and tyrosine (Y). Due to the inconsistency of this pattern, there is weak correlation between the frequency difference in single-plastic designs *versus* the frequencies in promiscuous designs (*r*^2^ = 0.37, *p* = 0.13). The arrangement of amino acids in the promiscuous peptides shows no obvious motifs or patterns of polar and non-polar residues (Fig. S8). Analyzing the interaction energies of amino acid types over all MD simulations for all peptides (Fig. S9) indicates that (1) the adsorbed conformation and the surrounding peptide residues strongly influence the interaction since the interaction energy for each amino acid:plastic pair varies several kcal mol^−1^ for all amino acids, (2) most amino acids have the same average interaction energy between the five plastics, with R, H, N, and Q being notable deviations, and (3) hydrophobic residues interact favorably with plastic, while hydrophilic or charged residues have neutral or unfavorable interaction energies. Analyzing the promiscuous peptides in Table 3 shows that the residues that form strong interactions differ between plastics (Fig. S10), suggesting that the amino acids that drive adsorption differ between plastics. This conclusion should be taken with caution given the simplifications of MM/GBSA calculations. In MD simulations, Δ*G* strongly correlates with van der Waals interactions for all plastics (Fig. 2E), indicating that this interaction is key for peptide affinity. This aligns with the enrichment of R, M, and W in the promiscuous peptides, since the bulky side chains of these amino acids can form strong van der Waals interactions with the plastic. Given the different chemistries of the five plastics (PE and PP are purely aliphatic, while PET, nylon, and PVC contain polar and/or aromatic groups), we also explored the role of electrostatic and solvent energies. While the contribution of these two energies to peptide adsorption differs dramatically among the five plastics (Fig. S11), Δ*G* does not correlate with either energy for any plastic. This occurs because the sum of the two terms is roughly constant due to a strong negative correlation between them (Fig. S12). This does not necessarily imply that electrostatic interactions do not matter; instead, they likely help define the adsorbed peptide conformations that have strong van der Waals interactions while avoiding unfavorable electrostatic interactions.

### Design of selective plastic-binding peptides

Our second goal was to design selective plastic-binding peptides, which could help discriminate between plastics and separate them in microplastic pollution. The specific goal was to design peptides that bind selectively to PP over PET (or PP-selective peptides, for short). These two plastics were chosen since their different chemistries make them a suitable test case to determine the feasibility of designing selective plastic-binding peptides. PP-selective peptides were designed by optimizing the predicted affinity score difference between PP and PET during SA.

**Fig. 3:**
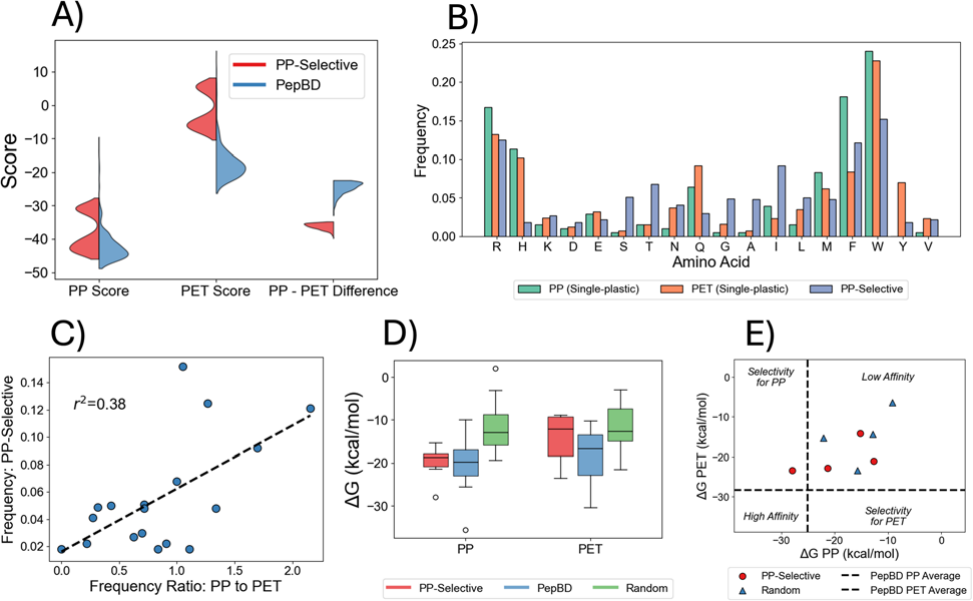
Design and evaluation of peptides that bind preferentially to polypropylene over PET. (A) Distributions of affinity scores for PP and PET, and the score difference between PP and PET. Results are shown for PP-selective and PepBD peptides, with each set containing 1000 peptides. (B) Amino acid composition of PP-selective designs and single plastic-binding designs for PP and PET. Data for each category was collected from the 1000 peptides with the best affinity scores. (C) Correlation between the ratio of amino acid frequencies in single-plastic designs for PP and PET *versus* the amino acid frequencies in the PP-selective designs. Ratios are calculated per the values in panel (B), dividing the value for PP by the value for PET. Each point corresponds to a different amino acid. (D) Δ*G* distribution of PepBD, PP-selective, and random peptides for PP and PET. Twelve peptides were tested in each category. PepBD peptides differ for each plastic. (E) Δ*G* from steered MD for PP-selective and random peptides. Black dashed lines indicate the average Δ*G* of four PepBD designs for PP and PET. Peptides affinity is classified as high if Δ*G* lies left of/below the horizontal/vertical black dashed lines. This divides the plane into four areas: selectivity for PP (high affinity for PP, low affinity for PET), selectivity for PET (low affinity for PP, high affinity for PET), high affinity (high affinity for PP and PET), and low affinity (low affinity for PP and PET).

The LSTM-SA model was able to identify peptides that have large, predicted selectivity for PP, primarily by reducing affinity for PET rather than by increasing affinity for PP. The 1000 best PP-selective peptides have a larger affinity difference between PP and PET than any PepBD peptides (Fig. 3A). Inspecting the affinity scores for either PP or PET shows that the PP-selective and PepBD peptides have similar predicted affinity for PP, but that the PP-selective peptides have much weaker predicted affinity for PET.

The amino acid composition of the PP-selective peptides differs notably from those of the single-plastic and promiscuous designs. The PP-selective peptides use low-mass amino acids (*e.g.* serine (S), threonine (T), glycine (G), and alanine (A)) at a much higher frequency (Fig. 3B). These amino acids were also uncommon in previous designs by PepBD^39^ and other ML models.^41,42^ Interestingly, SHAP analysis does not assign high importance to these amino acids, and instead assigns high importance to W and R (Fig. S7). We hypothesize that these low-mass residues allow W and R to interact more favorably with PP than PET. We also observe that amino acids that appear more often in single-plastic designs for PP than PET appear frequently in PP-selective peptides (Fig. 3C, *r*^2^ = 0.38, *p* = 0.0066). Such amino acids presumably interact more favorably with PP than PET, so this is a logical strategy for optimizing selectivity for PP.

The LSTM-SA model found multiple solutions for optimizing predicted selectivity for PP. Revisiting Fig. 3A, the unimodal distribution of affinity score differences between PP and PET contains a bimodal distribution of affinity scores for the individual plastics. Peptides in the two modes have different amino acid compositions and distinct arrangements of the amino acids (Fig. S13). Thus, these two modes appear to be distinct solutions for optimizing PP-selectivity. A third solution was inadvertently found when optimizing promiscuity. The promiscuous peptides have a large Δ*G* difference between PP and PET (Fig. 2B), and their amino acid composition differs from the PP-selective peptides' composition (Fig. S14).

MD simulations support the premise that the designed PP-selective peptides prefer PP over PET. Comparing Δ*G* of PP-selective, PepBD, and random peptides for the plastic PP (Fig. 3D) shows the PP-selective peptides have equal affinity as PepBD peptides and greater affinity than random peptides. The converse is true for PET:PP-selective peptides have worse affinity than PepBD peptides and equivalent affinity as random peptides. These results align with general selectivity for PP over PET, and four peptides with the largest selectivity are listed in Table 4. Analyzing the energetic contributions to Δ*G* shows that selectivity for PP arises not from a stronger intermolecular interaction energy (the sum of van der Waals, electrostatic, and GBSA solvation energies), but rather from a smaller reduction in conformational entropy upon adsorbing (Fig. S15). This is reflected by peptide residues not consistently forming stronger intermolecular interactions with PP than PET (Fig. S16). The conformational entropy was calculated using harmonic normal mode analysis,^63^ a large simplification for evaluating the conformational entropy of flexible molecules like peptides. Thus, we performed an additional evaluation of the four peptides in Table 4 using steered MD (see SI for details). Peptide affinity may be categorized as high or low by referencing the average Δ*G* of four PepBD designs for each plastic (see SI for sequences). One of the four PP-selective designs shows selectivity for PP (Fig. 3E). This peptide is bolded in Table 4. Comparing the distances of each amino acid of this design and either the PP or PET surface at the beginning of each SMD simulation (Fig. S17) indicates that M4, L9, I10, and W12 may be responsible for PP-selectivity, as they are more likely to be proximal to a PP surface than a PET surface. Random peptides may have sizable Δ*G* differences between PP and PET, but they all lie in the low affinity range and are thus not ideal for microplastic remediation.

**Table 4:** Example peptides with selectivity for polypropylene over PET

Sequence/description	Equilibrium MD results^a^			Steered MD results		
	Δ*G* PP ± 4.3 kcal mol^−1^	Δ*G* PET ± 4.8 kcal mol^−1^	Δ*G* PP − Δ*G* PET kcal mol^−1^	Δ*G* PP kcal mol^−1^	Δ*G* PET kcal mol^−1^	Δ*G* PP − Δ*G* PET kcal mol^−1^
Average random	−11.6	−11.9	0.3	−14.4	−14.9	−0.5
Average PepBD	−20.3	−17.9	N/A	−25.1	−28.4	N/A
NDLMFRRGLIFW (PP-selective)	−21.4	−9.2	−12.2	−15.2	−14.1	−1.1
FWWQQIGGNRQF (PP-selective)	−18.3	−8.9	−9.5	−12.7	−21.0	8.3
SNMMFRRGLIHW (PP-selective)	−17.8	−9.3	−8.6	−21.3	−22.8	1.5
**TAFMFRRGLIFW (PP-selective)**	**−21.2**	**−11.7**	**−9.5**	**−27.9**	**−23.4**	**−4.5**

a Uncertainty in Δ*G* calculated from the mean average error between two evaluations of the 12 random peptides performed separately for each plastic.

## Conclusion

We present an LSTM-SA model that designs peptides with promiscuous or selective binding to plastics. The LSTM is trained on biophysical data generated by PepBD to predict peptide affinity for one of five different plastics: PE, PP, PET, PVC, and nylon. Affinity predictions require only the peptide amino acid sequence, a feature shared by other recent ML models trained on PepBD data.^41,42^ This contrasts with PepBD and other biophysical modeling methods, which also require the peptide conformation when calculating peptide affinity. Since only sequence information is needed, the affinity of a given peptide for multiple plastics can be easily calculated simultaneously. This enables the optimization of peptide promiscuity or selectivity using SA. The resulting designs have significantly greater predicted promiscuity or selectivity than any of the millions of peptides in the PepBD dataset and are validated in MD simulations.

This work makes two practical contributions towards the development of peptide-based tools for microplastic remediation. First, the promiscuous plastic-binding peptides may simplify capture or detection of microplastic pollution. While we had previously discovered single plastic-binding peptides, remediating the multiple types of plastic typically found in microplastic pollution is more straightforward when using a promiscuous plastic-binding peptide. Second, the selective plastic-binding peptides could help detect specific plastics in microplastic pollution, separate microplastics into its components, or help plastic-degrading enzymes adsorb to the plastic they degrade. As noted in the Introduction, these peptides could augment existing methods for microplastic remediation.

This work explores how much peptide affinity varies between plastics. While the five plastics considered have different chemistries, the impact of these differences on peptide affinity is unclear. Do plastics differ sufficiently such that a peptide can distinguish between them? Our best PP-selective design (Table 4, bolded entry) and the sizable affinity differences shown by random peptides between PP and PET (Fig. 3E) suggests that this may be possible. Conversely, can a peptide bind strongly to several plastics despite their different chemistries? Our promiscuous plastic-binding peptides suggests that the answer is yes. The existence of both promiscuous and selective plastic-peptides reflects that the driving forces for adsorption may differ between peptides.

MD data supporting the promiscuity and selectivity of the peptides (Fig. 2 and 3) should be viewed with caution. For equilibrium MD simulations, calculation of Δ*G* relies on the generalized Born solvent model which treats the role of solvent approximately, and on normal mode analysis which has known limitations for evaluating conformational entropy for flexible biomolecules.^57^ The steered MD simulations build confidence in the best PP-selective design, but additional evaluation could still be useful. We did not perform SMD simulations for the promiscuous peptides due to the large computational cost – they require 2.5-fold more computer time as peptide affinity must be evaluated for five plastics rather than two. The best designs in Tables 3 and 4 could be evaluated first. Possible simulation methods for more rigorous free energy calculations include metadynamics^64^ or umbrella sampling.^65^ Experimental testing will be essential, especially for determining the degree of selectivity needed for a peptide to adsorb specifically to a given plastic, and the degree of promiscuity needed for a peptide to bind strongly to all types of plastic. We also note that our goal is not to perfectly predict Δ*G*, but instead to use computational predictions to minimize the effort of experimental validation. For example, even though not all design in Fig. 3E showed PP-selectivity, only 4 peptides needed to be tested before finding a successful design.

Our LSTM-SA model (Fig. 1A) can be extended to other peptide design goals. While we do not expect the model to be impactful in areas where ML has been developed extensively, such as peptide-protein binding^66^ or antimicrobial peptides,^67^ the model could be useful for the general problem of discovering solid-binding peptides. Such peptides have many uses in biotechnology and medicine.^16,17^*De novo* design of solid-binding peptides with ML relies on phage display data.^33,35^ Replacing qualitative phage display results with biophysical modeling data could give more useful and interpretable ML models. This approach will only work if peptide adsorption can be modeled accurately, which is not an easy task.^68,69^ Fortunately, there have been continual improvements in the past two decades in molecular force fields and modeling of solid interfaces, suggesting that this avenue of research could be fruitful.

## Author contributions

Conceptualization: FY, CKH. Methodology: VJ, MTB, CKH, FY. Investigation: VJ, MTB, CKH, FY. Software: VJ. Visualization: VJ, MTB. Supervision: FY, CKH. Writing—original draft: VJ, MTB. Writing—review & editing: VJ, MTB, CKH, FY.

## Note after first publication

This article replaces the version published on 1st October 2025, where Fig. 2 and 3 appeared identically, this has now been resolved.

## Conflicts of interest

There are no conflicts to declare.

## Abbreviations

PBP: Plastic-binding peptides
PE: Polyethylene
PP: Polypropylene
PET: Polyethylene terephthalate
PVC: Polyvinyl chloride
nylon: Nylon 6-6
LSTM: Long short-term memory network
MD: Molecular dynamics
SA: Simulated annealing
HTS: High throughput screening
MM/GBSA: Molecular mechanics/generalized Born surface area

## Supplementary Material

SC-OLF-D5SC04903B-s001

SC-OLF-D5SC04903B-s002

## Data Availability

The PepBD dataset and the LSTM model can be found on GitHub at https://github.com/PEESEgroup/LSTM-SA. Supplementary information: computational methods, supplemental figures and tables, and raw data files. See DOI: https://doi.org/10.1039/d5sc04903b.
