# Supplementary material for: AI-driven rational design of promiscuous and selective plastic-binding peptides
Source: Chem Sci. 2025 Oct 1;16(44):20823–32. doi: 10.1039/d5sc04903b (PMC12486148; doi:10.1039/d5sc04903b)
Supplement: SC-016-D5SC04903B-s002 [file SC-016-D5SC04903B-s002.pdf]

Supplementary Information (SI) for Chemical Science.
This journal is © The Royal Society of Chemistry 2025

Supplementary Information for

AI-Driven Rational Design of Promiscuous and Selective Plastic-Binding Peptides

Vinamr Jain[1‡], Michael Bergman[2‡], Carol Hall[2], Fengqi You[*1,3,4,5]

[1] College of Engineering, Cornell University, Ithaca, New York 14853, USA

[2] Department of Chemical and Biomolecular Engineering, North Carolina State University, Raleigh, NC 27606, USA

[3] Robert Frederick Smith School of Chemical and Biomolecular Engineering, Cornell University, Ithaca, New York 14853, USA

[4] Cornell University AI for Science Institute, Cornell University, Ithaca New York 14853, USA

[5] Cornell AI for Sustainability Initiative (CAISI), Cornell University, Ithaca New York 14853, USA

[*]Corresponding author: Fengqi You (fengqi.you@cornell.edu)

Methods

PepBD Dataset for polyethylene, polypropylene, and PET

The PepBD dataset used to train the LSTM score predictors for PE, PP, and PET was taken from previous work[1]. We briefly summarize PepBD here for convenience. PepBD uses simulated annealing to search peptide amino acid sequences and peptide conformations and identify peptides with high predicted affinity for a given plastic. Peptide affinity for plastic is measured via the PepBD score, which is the sum of the peptide-plastic interaction energy and a weighted peptide internal energy. The interaction energy and internal energy are both calculated using the molecular mechanics/generalized Born surface area (MM/GBSA)[2]. A lower PepBD score corresponds to higher predicted affinity. Designs are performed separately for each plastic, with between 35 to 100 design runs performed per plastic. Different adsorbed structures are used to initiate PepBD to increase sampling of peptide adsorbed conformations. Results from all design runs were aggregated to create the PepBD dataset. If an amino acid sequence was sampled multiple times, either in one design run or across multiple design runs, only the most negative score was retained. The resulting PepBD dataset contains 715,509 sequences for PE, 433,488 sequences for PP, and 441,978 sequences for PET. All peptides are 12 residues and may contain any of the 20 natural amino acids except cysteine and proline.

Generating the PepBD Datasets for Nylon 6-6 and PVC

New PepBD designs were performed in this work for nylon 6-6 (nylon) and PVC as PepBD had not previously been performed for these plastics. Atomic partial charges of PVC and nylon monomers were calculated for a 3-monomer chain with the RED webserver[3], then selecting the partial charges from the internal monomer. All other parameters (Lennard Jones parameters, bond angles, bond lengths, etc.) were taken from the GAFF2 forcefield[4]. A rectangular plastic surface of dimensions 8 nm by 8 nm by 2 nm was created per our previous process[1]. An ensemble of adsorbed peptide conformations on each plastic surface, which is needed to initiate PepBD design, was obtained by simulating an arbitrary peptide (KWFFEKWWMMRR) in explicit solvent at 500K for 100 nanoseconds in a simulation box size of 8 nm by 8 nm by 5 nm. The simulations applied an UPPER WALL with PLUMED so the distance between the peptide center of mass and the top surface of the plastic did not significantly exceed 2 nm, thereby preventing the peptide from diffusing too far from the surface. Other simulation parameters are described below in

“Evaluating ΔG in Equilibrium and Steered Molecular Dynamics”. Thirty unique conformations were extracted from the simulation using k-means clustering with CPPTRAJ. A random member of each cluster was used to initiate PepBD design, and three PepBD design runs were performed per conformation. Collecting all unique sequences sampled by PepBD for each plastic gave the PepBD dataset with 208,609 peptides for PVC and 142,615 peptides for nylon.

LSTMs Affinity Score Predictors

To predict the PepBD scores, we employed a Long Short-Term Memory (LSTM)-based regression model, leveraging its ability to effectively capture the sequential dependencies inherent in peptide sequences. LSTMs have been widely adopted in sequence modeling tasks due to their capacity to mitigate vanishing gradient issues while preserving long-range dependencies[5].

The proposed architecture consists of two LSTM layers, each with a hidden dimension of 512, followed by a hyperbolic tangent (Tanh) activation function and a final linear output layer. The PepBD scores were normalized using a standard scaler (by subtracting the mean and dividing by standard deviation across the dataset) to ensure numerical stability during training. The model was optimized using the AdamW optimizer[6], which integrates weight decay regularization to mitigate overfitting. Training was performed by minimizing the Mean Squared Error (MSE) loss function with a learning rate of 0.001 and a batch size of 32.

To prevent overfitting, early stopping was implemented with a patience of 10 epochs, leading to an effective training duration of approximately 60–80 epochs per experiment (Fig. S3). Early stopping is a widely used regularization technique that halts training when validation performance ceases to improve, thereby reducing unnecessary computation and mitigating overfitting[7]. Additionally, a dropout rate of 0.1 was applied to the LSTM layers to enhance model generalizability by preventing co-adaptation of neurons[8].

All experiments were conducted using an 80/10/10 train-validation-test split. Model training was performed on a high-performance workstation equipped with an NVIDIA GeForce RTX 3080 GPU (10GB RAM) and an Intel® Core™ i9-10920X CPU (24-Core, 3.50 GHz). Upon training completion, the predicted scores were rescaled to match the original range of the PepBD dataset. A comparative analysis of different recurrent network architectures was also conducted, including bidirectional LSTMs (BiLSTM), Gated Recurrent Units (GRUs), vanilla RNNs and

Transformers (Table 1) for the peptide affinity score prediction task. Table S1 summarizes the model architectures along with their respective trainable parameters.

Peptide Design with Simulated Annealing

We utilize SA to optimize peptide sequences for enhanced binding affinity, following the general optimization strategy previously employed in generating the PepBD dataset[1]. However, our approach significantly differs from the original methodology by substituting computationally intensive sampling of peptide structures and binding energy calculations with rapid predictions from a surrogate LSTM neural network that predicts PepBD affinity scores from peptide sequence alone.

To illustrate, PepBD sampling requires approximately 3.25 seconds to sample one peptide sequence, equating to around 26.6 hours per optimization run (sampling roughly 28,800 sequences per run on an Intel® Xeon® Gold 6130 CPU). In contrast, our surrogate-driven approach, implemented on an Intel® Core™ i9-10920X CPU (24-core, 3.50 GHz), significantly reduces runtime ($\approx$ 0.01s to sample a peptide sequence) by avoiding structural calculations, enabling more extensive sampling within a smaller computational timeframe (Table 2).

The optimization process begins by selecting initial peptide sequences that undergo iterative random mutations. Two types of mutations are applied: amino acid substitutions, where one randomly chosen residue is replaced, and amino acid swaps, where residues at two random positions are interchanged. Mutation probabilities are set at 75% for substitutions and 25% for swaps. Each mutated sequence is then one-hot encoded and evaluated by the surrogate LSTM model to estimate its affinity score.

Acceptance of each sequence modification follows the Metropolis criterion, which probabilistically accepts or rejects changes based on predicted affinity scores. The acceptance probability P_{acc} is calculated as:

$$P_{\text{acc}} = \min\left(1, \exp\left(-\frac{\widehat{Y_{new}} - \widehat{Y_{old}}}{kT}\right)\right)$$

where $\widehat{Y_{\text{new}}}$ and $\widehat{Y_{\text{old}}}$ represent predicted affinity scores for the modified and original sequences, respectively, and kT (Boltzmann constant multiplied by temperature) regulates exploration. The selected parameters for the initial temperature and cooling ratio were adapted from PepBD's original settings, modified slightly to encourage conservative yet thorough exploration of peptide

sequence space. In this study, The SA algorithm utilizes a temperature schedule starting at a high temperature (kT_{start} = 2) to facilitate broad exploration and progressively decreases to a minimum temperature (kT_{end} = 0.1). The temperature is lowered every 75 iterations by a constant ratio (r = 0.99), gradually reducing acceptance of sequences with poorer predicted affinity scores. This systematic cooling balances exploratory and exploitative search strategies, guiding convergence towards peptides with optimal affinity scores. For each design goal, the SA optimization initiated from 55 randomly selected peptide sequences and performed 22,525 iterations per run to generate peptide candidates.

Our framework extends beyond single-plastic affinity optimization to design promiscuous- or selective plastic-binding peptides. For promiscuous plastic-binding peptides, the objective function was defined as the average predicted affinity across the considered plastics:

$$\hat{Y} = \frac{\widehat{PET} + \widehat{PE} + \widehat{PP} + \widehat{PVC} + \widehat{NYL}}{5}$$

For selective plastic-binding peptides, the optimization function was the difference in affinity between the targeted plastic and a non-target plastic, exemplified by polypropylene-selective peptides avoiding polyethylene terephthalate (PP-PET selectivity):

$$\hat{Y} = \widehat{PP} - \widehat{PET}$$

SHAP analysis of Promiscuous and Selective Designs

We employed SHAP (SHapley Additive exPlanations) analysis with a GradientExplainer to quantify the contribution of individual amino acid residues and their positions to the final trained LSTM models' binding affinity predictions. Peptide sequences were represented using one-hot encoding. To ensure comparable attributions across different objectives and models, we constructed a shared, plastic-agnostic background distribution from 200 uniformly sampled 12-residue sequences drawn from the same amino acid set used in the PepBD datasets (all natural amino acids except cysteine and proline).

We analyzed the design results for our two objectives:

1. Promiscuous binding peptides: A composite predictor was defined as the mean of predictions from all five plastic-specific LSTM models. Prior to aggregation, model outputs were inverse-transformed from their training normalization to the original PepBD score values to ensure interpretability. SHAP attributions were computed both on the

composite predictor and separately on each individual plastic-specific model. The composite attributions were then compared to the individual plastic-specific attributions to assess consistency across models.

2. PP-selective peptides: The selectivity predictor was defined as the difference between polypropylene and PET model predictions of PepBD scores. SHAP attributions were computed for this difference function and separately compared to individual attributions for PP and PET models.

SHAP attributions were aggregated at two hierarchical levels to capture different aspects of feature importance

1. Position importance: the sum of attributions across all amino acid channels at each of the 12 residue positions
2. Amino acid importance: the sum of attributions across all sequence positions for each of the 18 amino acids.

To align SHAP values with binding affinity interpretation, we multiplied all attributions by -1 so that positive values indicate features that enhance binding affinity (lower PepBD scores), while negative values indicate features that reduce binding affinity (higher PepBD scores), where enhancement and reduction are both with respect to the random peptide baseline. Averages and standard deviations were calculated for the importance scores of each dataset (the top 1000 promiscuous or the top 1000 PP-selective peptides).

Evaluating ΔG in Equilibrium and Steered Molecular Dynamics

Equilibrium MD simulations were performed to evaluate peptide affinity for plastic per the procedure used in previous work[10–12], which we summarize here. Each peptide:plastic combination undergoes many equilibrium simulations to evaluate the adsorption energy of a peptide to a plastic over several different adsorbed conformations. The ensemble of adsorbed conformations was obtained by simulating the peptide:plastic system at 550K, where the high temperature results in rapid sampling of different adsorbed peptide conformations. Sixteen different adsorbed conformations were extracted via k-means clustering with CPPTRAJ[13]. Each adsorbed conformation was evaluated by equilibrating at 300K then running a 1 ns simulation in the NVT ensemble. The MM/GBSA (molecular mechanics/generalized Born solvent-accessible area) tool of Amber was used to calculate ΔG for all conformations. We use the GB implicit solvent model

rather than the Poisson-Boltzmann implicit solvent model since one model never has greater predictive accuracy than the other, the GB model is computationally cheaper, and only the GB model can be used when calculating the entropy via harmonic mode analysis with AmberTools. That being said, in the future we will explore the sensitivity of peptide ranking on the implicit solvent model. The 8 conformations with the lowest simulated ΔG were simulated an additional 4 ns in the NVT ensemble before recalculating ΔG, now including an estimate for the change in conformation entropy using harmonic normal mode analysis[14]. Peptide affinity for a plastic was then set to the lowest ΔG, as this is the most stable conformation out of the sampled states.

Steered MD (SMD) simulations were performed to more rigorously evaluate ΔG_{ads} of the PP-selective peptides. SMD was performed using PLUMED[15], with the collective variable defined as the distance between peptide center of mass and the top of the plastic surface, which we name z_{pep}. A diverse set of adsorbed conformations for each peptide:plastic pair to initiate SMD was provided by the 16 conformations obtained during equilibrium MD simulations. The system was solvated so the water layer extended at least 5 nm above the plastic surface. Each system was equilibrated by performing NVT equilibration for 100 ns, then NPT equilibration for 200 ps. During equilibration, a harmonic constraint was applied to z_{pep} to prevent desorption. SMD was performed by applying time-dependent external bias (in the form of a harmonic potential whose minimum is varies with time) the collective variable, which induces the peptide to desorb. At the start of the simulation, the bias minimum was the initial distance after equilibration. The bias minimum was moved at 0.5 nm/ns for 5 ns, giving a total displacement of 2.5 nm. The work performed by the bias over the simulation can be by the product of the external force and the displacement of the bias energy minimum. The total work over the full simulation is given by the sum over all time intervals. This six times per each starting conformation, giving a total of 96 simulations per peptide. The ΔG_{ads} value was calculated from the corresponding distribution of work values using the Jarzynski relation[16], $e^{-\frac{\Delta G_{ads}}{RT}} = \langle e^{-\frac{W}{RT}} \rangle$, where, T is the system temperature, R is the gas constant, W is the work performed by the bias, and the operator $\langle \cdot \rangle$ denotes an average.

MD simulations were prepared using tLEaP[17] in Amber, converted to Gromacs format using Parmed[13], and run with Gromacs version 2019.6[18]. Bonds to hydrogen were constrained using LINCS[19]. Long-range electrostatic interactions were treated using the particle mesh Ewald method[20]. Temperature in the NVT and NPT ensemble was controlled using the velocity scaling thermostat[21], with separate thermostats for water and non-water atoms. Pressure in the NPT

ensemble was controlled using the Berendsen barostat[22] with a time constant of 5 ps, semi-isotropic coupling so that the simulation box dimension normal to the plastic surface could change independently of the dimensions parallel to the surface, and an isothermal compressibility of 4.5 $\times 10^{-4}$ in all three directions. The simulation time step was 2 fs. Water was represented with the TIP3P model[23], plastics were represented with GAFF[4] and partial charges taken from previous work[1], and peptides were represented with the ff14SB force field[24]. Atomistic models of PE, PP, and PET were taken from previous work[1]. Position restraints with a force constant of 5000 kJ/mol/nm^2 were added to all carbon atoms in the plastic to maintain the rectangular geometry of the plastic surface during all simulation stages.

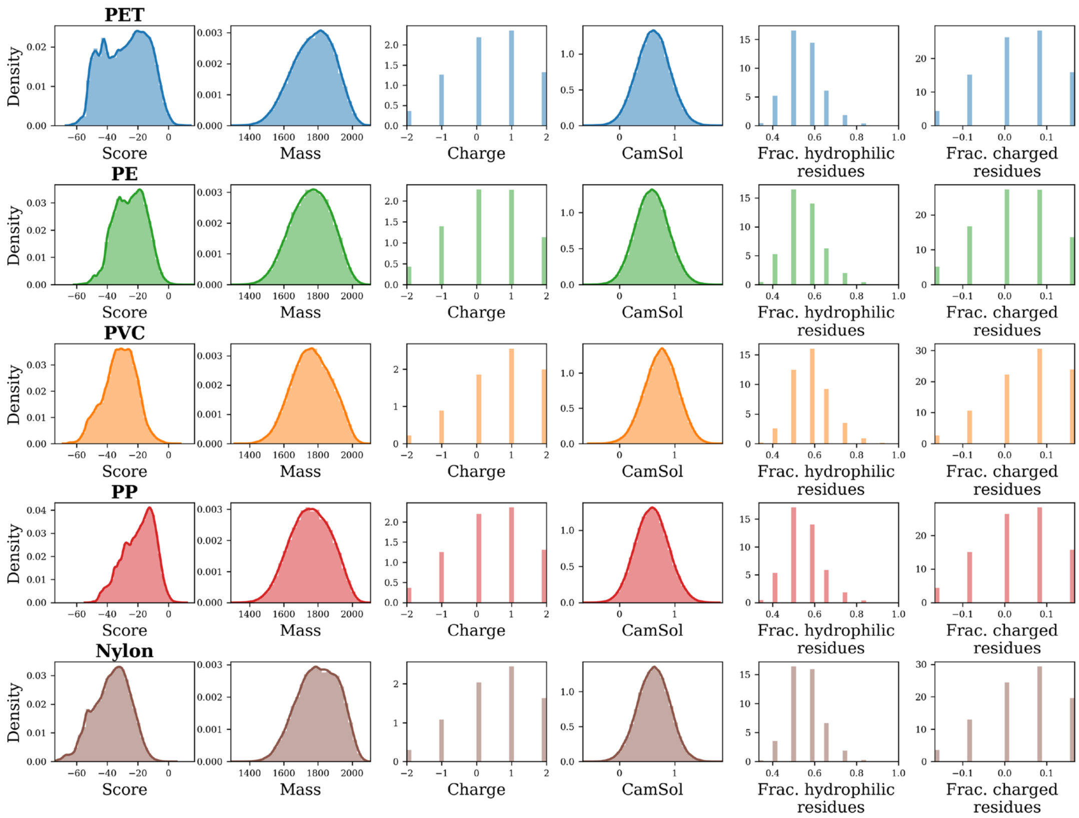

Figure S1. PepBD Score and physicochemical property distributions of the training datasets across five plastic types. Distribution plots showing (left to right): PepBD affinity scores, molecular mass, net charge, CamSol solubility, fraction of hydrophilic residues, and fraction of charged residues for peptides in the training datasets for PET, PE, PVC, PP, and Nylon. Each row represents a different plastic substrate, with datasets containing 441,978 (PET), 715,509 (PE), 208,609 (PVC), 433,488 (PP), and 142,615 (nylon) peptide sequences, respectively.

Fig. S1 compares the physicochemical property distributions of peptides in the PepBD training datasets for each plastic. All datasets show similar molecular mass distributions centered around 1600-1800 Daltons and comparable charge distributions slightly favoring neutral to positively charged sequences. However, the location and shape of the affinity score distributions vary significantly between plastics. The score distribution for nylon has the most favorable (negative) scores and a broad distribution, while PE and PP exhibit higher (less favorable) average scores. The distribution of CamSol values indicates that the peptides span from low to medium to high predicted aqueous solubility across all plastics. The fraction of hydrophilic and charged residues is consistent between plastics.

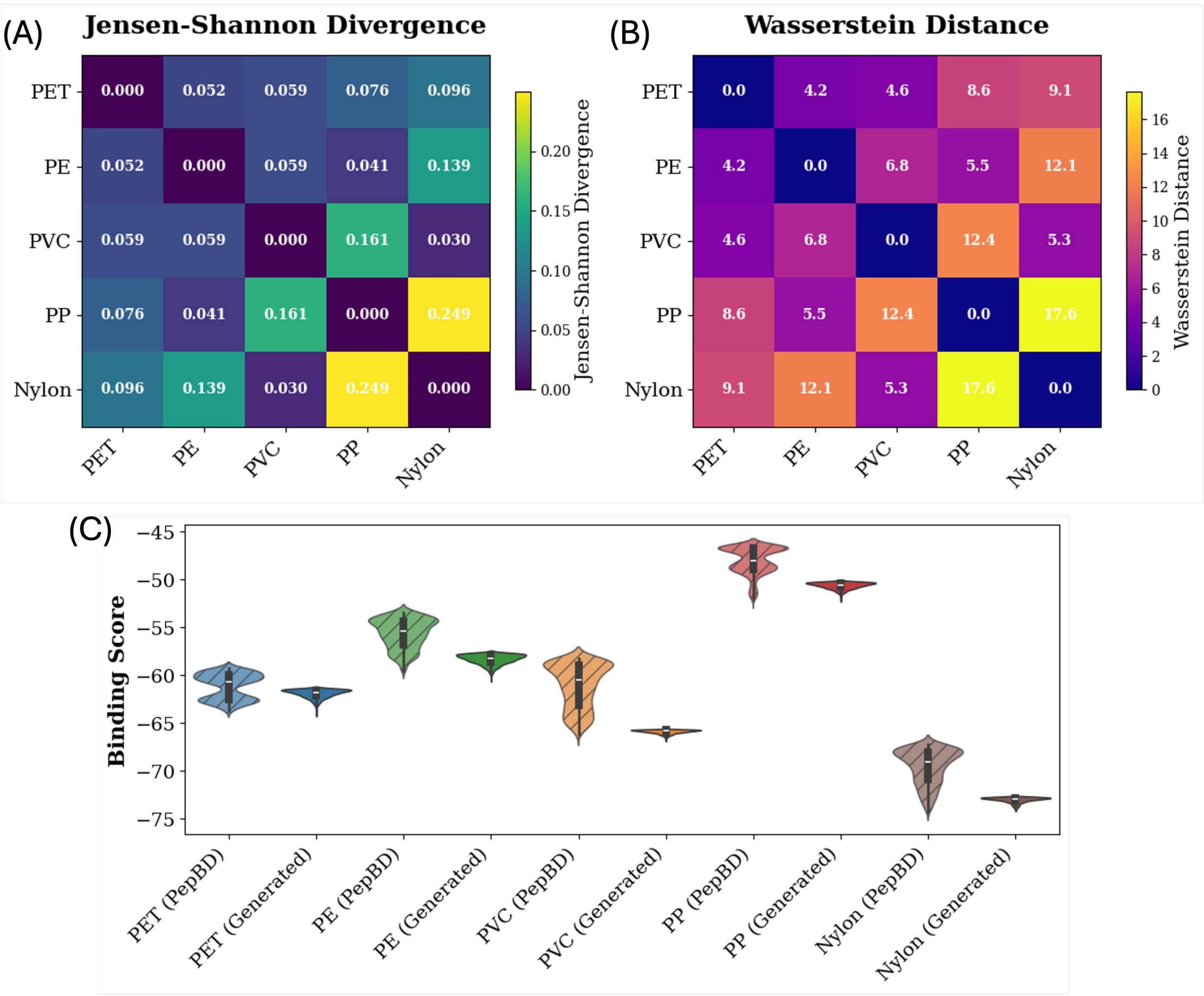

Figure S2. Comparison of PepBD datasets and generated sequences. (A) Jensen-Shannon divergence heatmap showing score distributional differences between the PepBD datasets (PET, PE, PVC, PP, Nylon). Lower values (darker colors) indicate greater similarity between datasets. (B) Wasserstein distance heatmap quantifying the distributional distance between the same plastic datasets, with higher values (warmer colors) representing greater dissimilarity. (C) Violin plots comparing binding score distributions of the top 1000 PepBD peptides versus the top 1000 generated peptides optimized for each specific plastic type using the LSTM-SA pipeline.

Panels A and B of Fig. S2 compare the score distribution difference of the PepBD datasets across the different plastics, using quantitative divergence metrics. The pairs PP-Nylon and PP-PVC have a high distributional difference, PE-Nylon are moderately different, and other plastic pairs are similar. These scores are normalized using a standard scaler before being passed on to the LSTM

layer while training for score prediction and then rescaled back during inference. Panel C of Fig. S2 shows a comparison between the score distributions between top candidates from the PepBD datasets and the generated sequences using the LSTM-SA pipeline, optimized for the individual plastics. This can be seen by the concentration of the generated sequences towards lower scores (higher affinities).

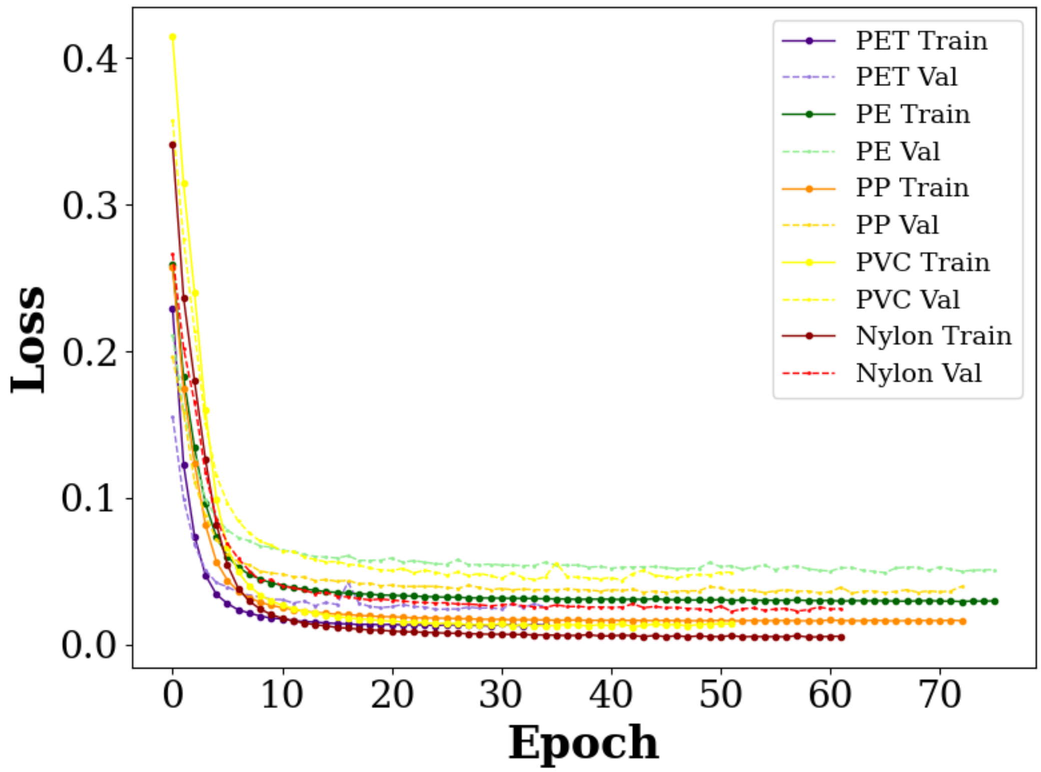

Figure S3. Training and validation loss curves for LSTM models trained on different plastic datasets, with early stopping implemented. Model convergence typically occurred within 60-80 epochs.

Fig. S3 shows the training and validation loss curves when fitting LSTM score prediction models for each of the five plastics. To mitigate overfitting, we implemented an early stopping mechanism with a patience parameter of 10 epochs. This approach automatically terminated training when no improvement in validation loss was observed for 10 consecutive epochs, resulting in effective training durations of approximately 60-80 epochs across all experiments.

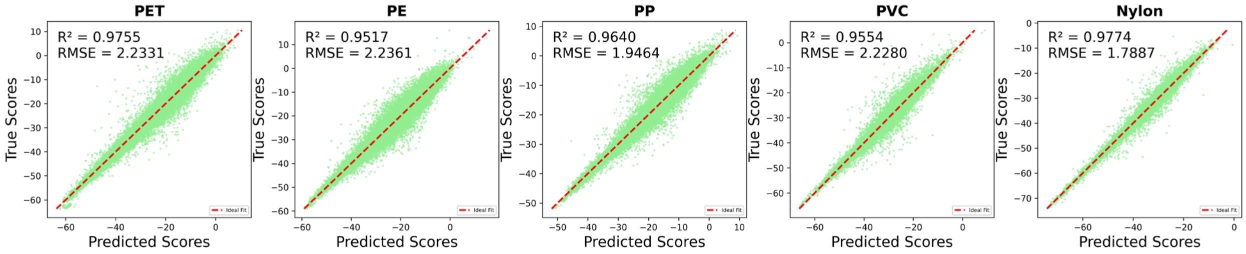

Figure S4. Predicted vs. ground truth affinity scores from the LSTM model across the test set for different plastic types. The model exhibits strong agreement with ground truth values, indicating high predictive reliability across all plastic substrates.

Fig. S4 shows validation scatter plots that illustrate the relationship between predicted and actual affinity scores across test set splits for different plastic types. The LSTM achieves high predictive accuracy with low error margins (RMSE ≈ ±2), reflected by coefficients of determination (R^2) ranging from approximately 0.95 to 0.97.

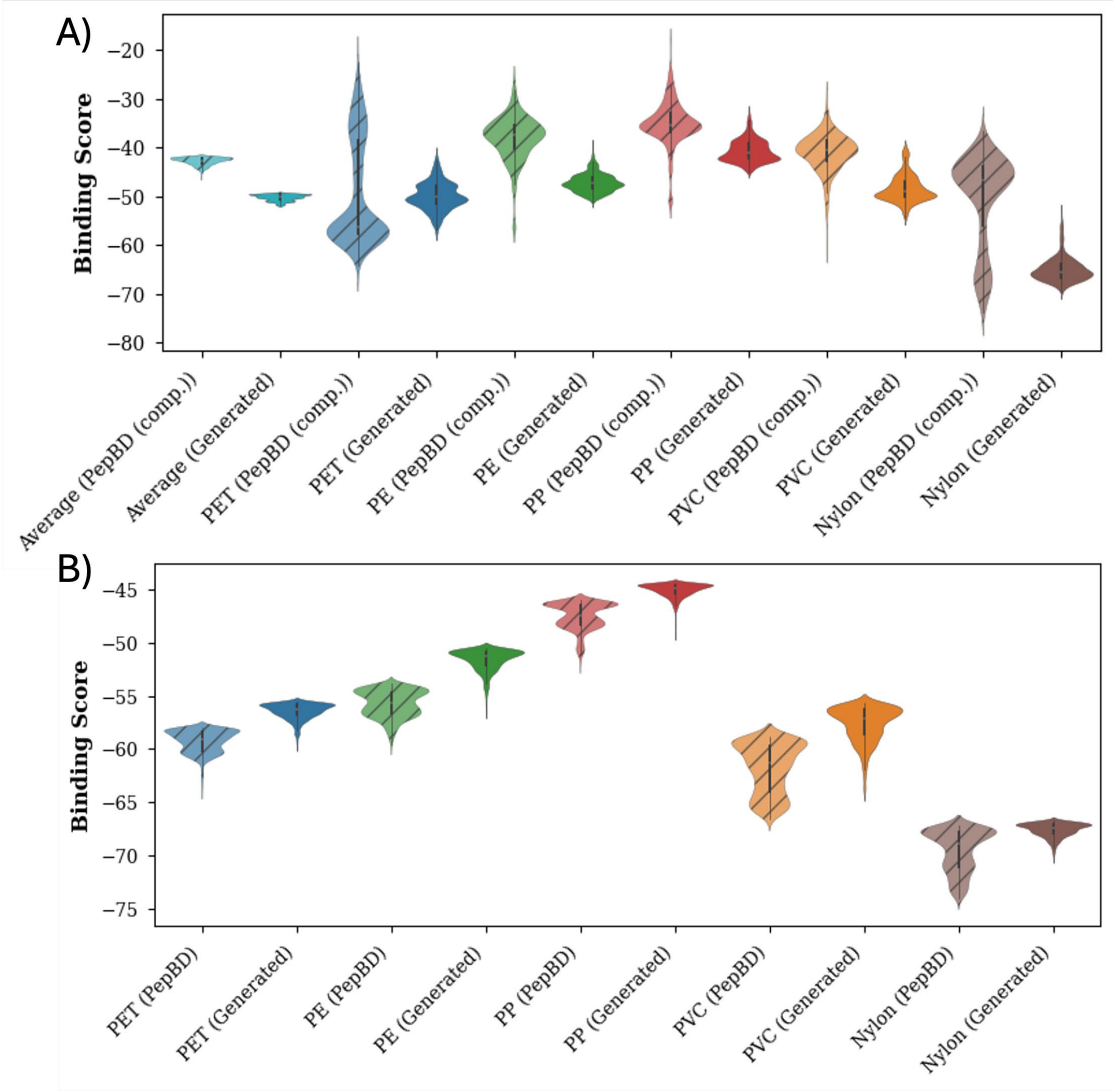

Figure S5. Binding Score Distribution of Promiscuous Peptides Generated by LSTM-SA. (a) Violin plot comparing predicted binding score distributions across different plastics between PepBD peptides and promiscuous plastic-binding peptides generated by LSTM-SA. Each dataset contains 1000 sequences. PepBD peptides were selected by compiling affinity scores for all peptides in the training data across all plastics, predicting scores with the LSTM model, and selecting the 1000 peptides with the highest average affinity scores. (b) Violin plot comparing predicted binding score distributions across different plastics between the top 1000 promiscuous

plastic-binding peptides generated by LSTM-SA and the top 1000 PepBD peptides selectively designed for each individual plastic.

Analysis of the affinity scores for individual plastics in Fig. S5 reveals that LSTM-SA generated peptides exhibit lower average affinity scores compared to PepBD comparative peptides for most plastics, with PET being the exception (Fig. S5A). Although these LSTM-SA peptides demonstrate the desired feature of binding to multiple plastics simultaneously, they show reduced affinity for each individual plastic when compared to PepBD peptides specifically optimized for those plastics (Fig. S5B). This observation highlights the inherent trade-off in multi-objective optimization, where optimizing for binding across multiple targets necessarily compromises the maximum achievable affinity for any single target.

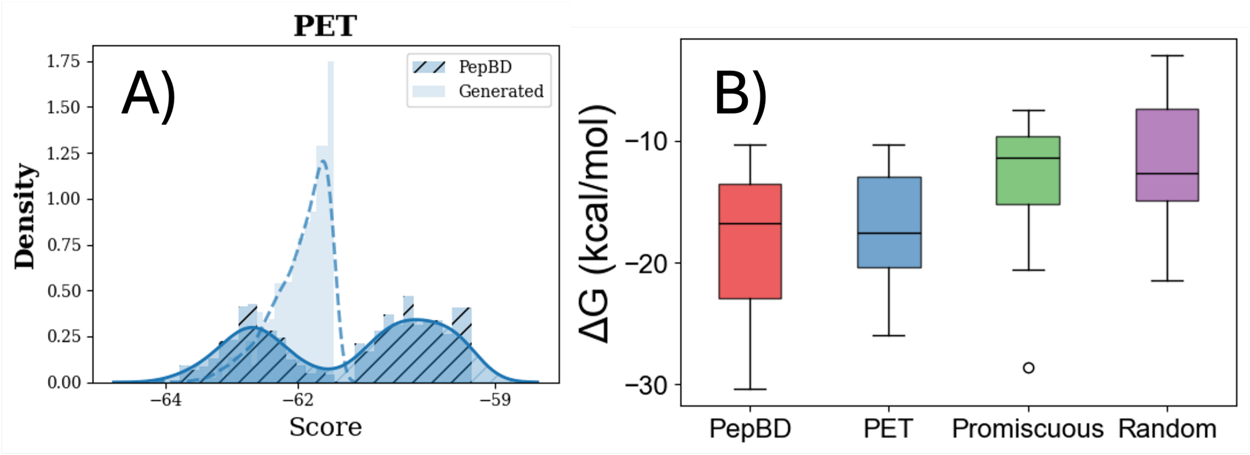

Figure S6. Affinity of peptides generated by the LSTM-SA to bind to PET. (a) Distribution of the predicted affinity scores. (b) Distribution of ΔG values from MD simulations. Results are shown for the best PepBD peptides, PET peptides generated by the LSTM-SA, promiscuous plastic-binding peptides generated by the LSTM-SA, and Random peptides.

Fig. S6 plots the affinity scores (panel A) and ΔG values from MD simulations (panel B) for peptides generated by the LSTM-SA when optimizing solely for PET affinity. For comparison, the two panels also include data for the best PepBD peptides, the promiscuous plastic-binding peptides generated by the LSTM-SA, and Random peptides. Whereas the promiscuous plastic-binding peptides have significantly lower affinity in both metrics compared to PepBD peptides, the PET peptides generated by the LSTM-SA have equivalent affinity as the PepBD peptides. This demonstrates that the LSTM-SA can generate peptides with high affinity for PET, but optimizing promiscuous plastic-binding affinity causes PET affinity to decrease significantly.

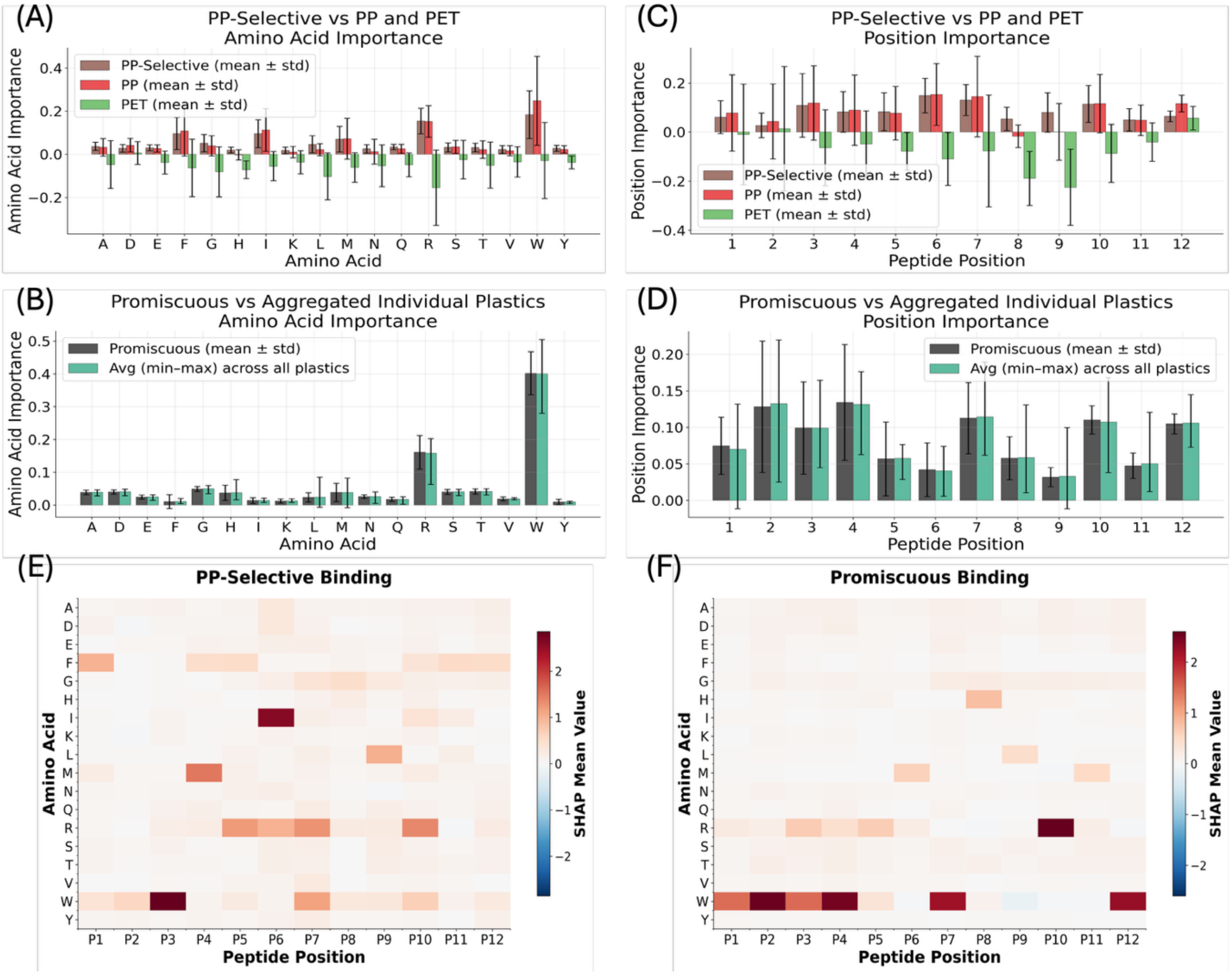

Figure S7. SHAP analysis of position and amino acid importance for PP-selective and promiscuous plastic-binding peptides. (A) Amino acid-specific SHAP importance scores for PP-selective peptides, comparing scores for PP-selectivity, PP, and PET. (B) Amino acid- specific SHAP importance scores for promiscuous peptides, comparing promiscuous scores to the aggregated average across all plastic types. (C) Position-specific SHAP importance scores, comparing scores for PP-selectivity, PP, and PET. (D) Position-specific SHAP importance scores, comparing promiscuous scores to the aggregated average across all plastic types. All values (means, standard deviations, and error bars) are normalized by the absolute sum of their respective group means. Error bars represent ± 1 standard deviation, with min-max ranges shown for comparisons across plastics in panels B and D. Heatmap of SHAP importance scores for the entire feature matrix corresponding to the amino acids and positions, averaged over all the sequences is shown in (E) for PP-selective and (F) for promiscuous peptides.

Fig. S7 provides a SHAP analysis of both the amino acid position and amino acid type for promiscuous and PP-selective designs. The SHAP analysis reveals that tryptophan (W) and arginine (R) are important contributors to promiscuous and PP-selective peptides. For PP-selective designs, phenylalanine (F), isoleucine (I), and methionine (M) also are attributed high importance. R appears especially important for PET binding, showing strong negative importance for PET binding and positive importance to PP-PET selectivity. For promiscuous peptides, the importance scores of amino acids closely match the score averaged over the individual plastics, as expected given that the composite predictor averages across plastic-specific models. The SHAP scores for positions may show great variation for PP-selective and promiscuous designs, with different patterns between the two design cases.

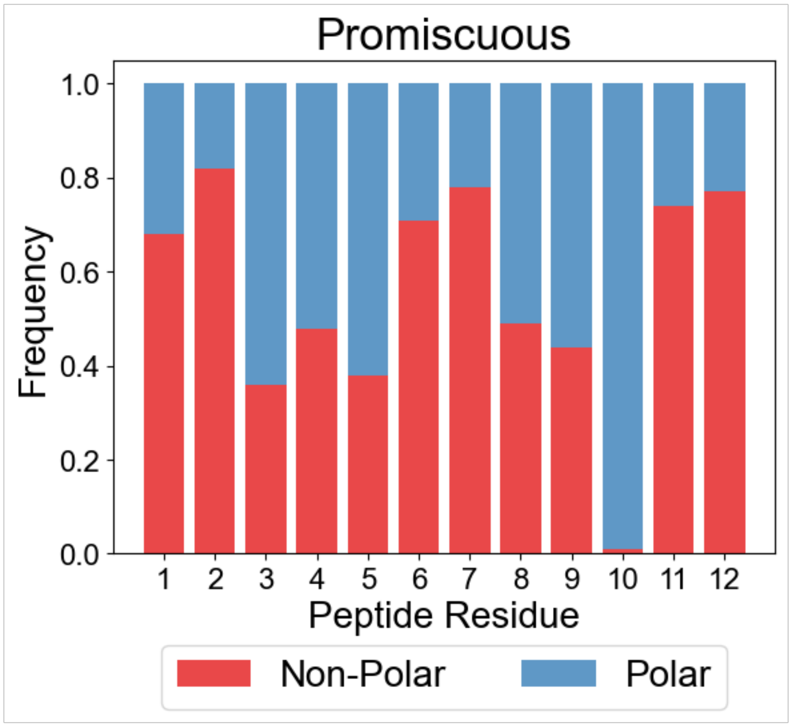

Figure S8. Patterning of polar and non-polar amino acids in the 100 promiscuous plastic-binding peptides with the best scores.

Fig. S8 shows the arrangement of non-polar residues (ILE, LEU, MET, PHE, TRP, TYR, and VAL) and polar residues (all other amino acids) in the 100 LSTM-SA promiscuous plastic-binding peptides with the best optimization scores. There is no notable pattern in the organization of non-polar and polar residues, other than a slight preference for non-polar residues at the termini and all peptides putting a polar residue at position 10.

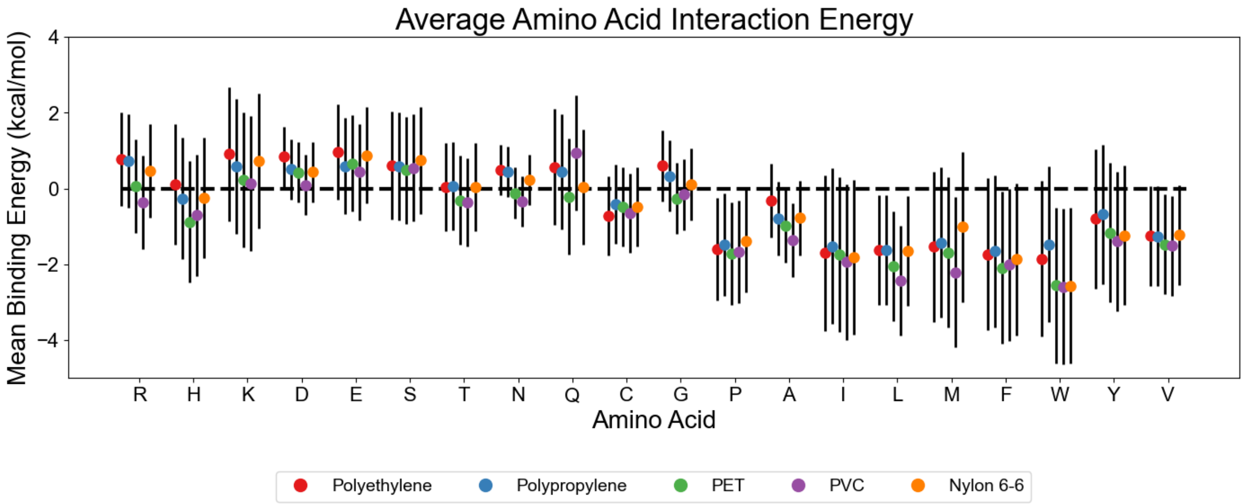

Figure S9. Average MM/GBSA binding energy of each amino acid type for each of the five plastics. Data collected for PepBD designs (unique designs for each plastic), Random peptides, and promiscuous peptides. Dots represent the mean, and bars represent ± 1 standard deviation.

Fig. S9 compares the interaction energy for each amino acid type over all peptides evaluated in MD simulations (i.e., PepBD, Random, or promiscuous peptides). Comparing averages between plastics can indicate if a certain amino acid interacts more strongly with one plastic versus another. The results show that 1) the interaction energy for each amino acid : plastic pair varies by several kcal/mol for all amino acids, indicating that the interaction energy depends strongly on the adsorbed conformation and the surrounding peptide residues, 2) most amino acids have the same average interaction energy between the five plastics, with R, H, N, and Q being notable deviations, and 3) hydrophobic residues interact favorably with plastic, while hydrophilic or charged residues have neutral or unfavorable interaction energies.

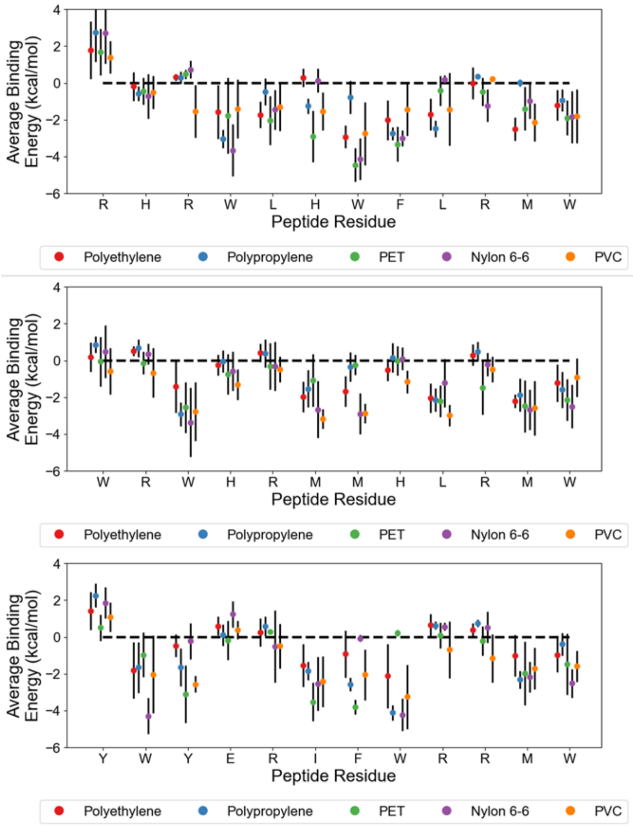

Figure S10. Per-residue interaction energy with each plastic for the most promising promiscuous peptides. The peptides analysed are taken from Table 3. Red, blue, green, purple, and orange circles indicate average interaction energy from the 8 simulations of each peptide adsorbing to polyethylene, polypropylene, PET, Nylon 6-6, and PVC, respectively. Black bars indicate ± 1 standard deviation from the 8 simulations for each peptide : plastic pair.

Fig. S10 compares the interaction energy for each residue for the most promising promiscuous peptides listed in Table 3. This analysis can help identify amino acids that contribute equally or disparately towards peptide affinity of the different plastics. While the average and range of interaction energies across the five plastics is similar for most amino acids, each peptide has 3 to 5 amino acids that vary greatly between plastics. Residues showing variability also tend to interact strongly with plastic (i.e., negative energies), suggesting that different residues drive adsorption of promiscuous peptides to different plastics. This conclusion should be taken with caution given the approximations in the MM/GBSA calculations used in the analysis.

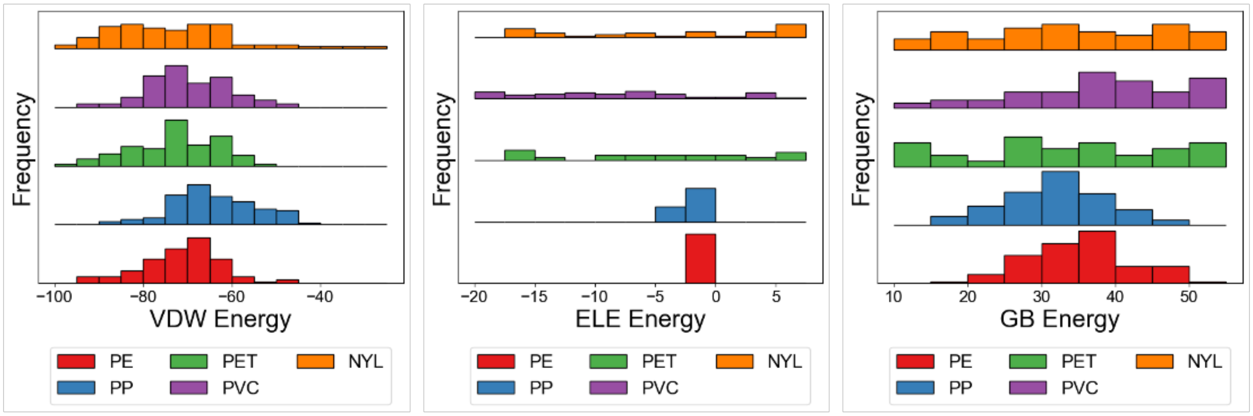

Figure S11. Energy components of peptide adsorption energies to the five plastics. Distribution of van der Waals (VDW), electrostatic (ELE), and generalized born solvent energy (GB) for all LSTM-SA promiscuous plastic-binding peptides after the final stage of equilibrium MD simulations. This analysis corresponds to the peptides described in Fig. 2 of the main text.

Fig. S11 plots the distribution of van der Waals (VDW), electrostatic (ELE), and generalized born solvent energy (GB) energies of the designed promiscuous plastic-binding peptides to five plastics: polyethylene (PE), polypropylene (PP), polyethylene terephthalate (PET), polyvinyl chloride (PVC), and nylon 6-6 (NYL). This data allows us to see how peptide-plastic interactions vary between the different plastics. The most notable difference regards electrostatic interactions, which are essentially non-existent for PE and PP, but are significant and typically favor peptide adsorption for PVC, PET, and NYL.

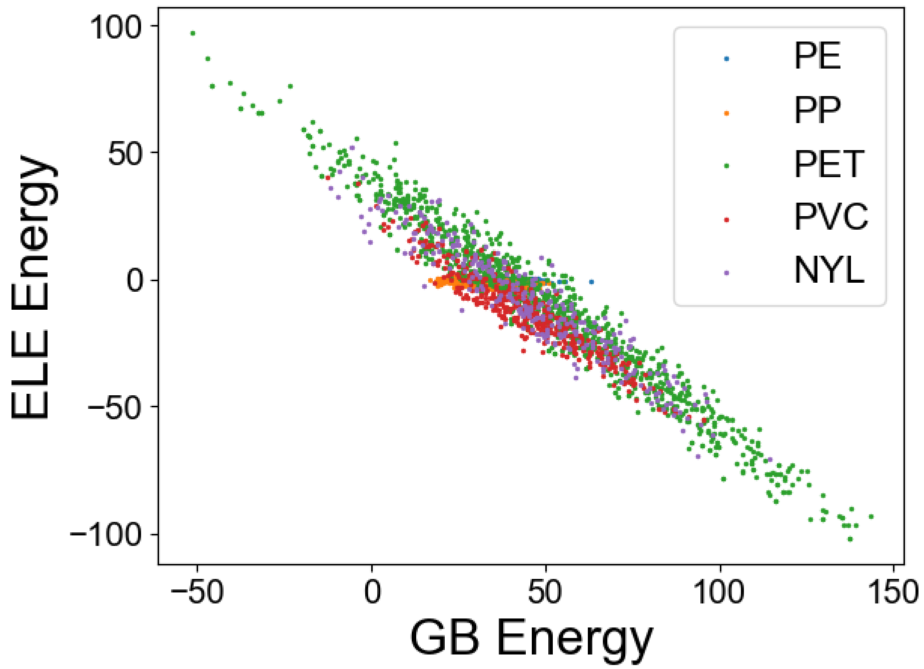

Figure S12. Correlation between electrostatic (ELE) and generalized Born polar solvation energy (GB) energies. Results were collected from all molecular dynamics (MD) simulations of PepBD, Random, and promiscuous plastic-binding peptides.

Fig. S12 plots the correlation between the electrostatic (ELE) and generalized Born polar solvation energy (GB) energies in molecular dynamics (MD) simulations. Two energies display a strong negative correlation, and all plastics appear to follow the same linear fit. The implication is that increasing the favorability of peptide-plastic electrostatic interactions decreases the favorability of peptide-water or plastic-water interactions. This occurs because peptide adsorption reduces the area of both the peptide and the plastic that can interact with water.

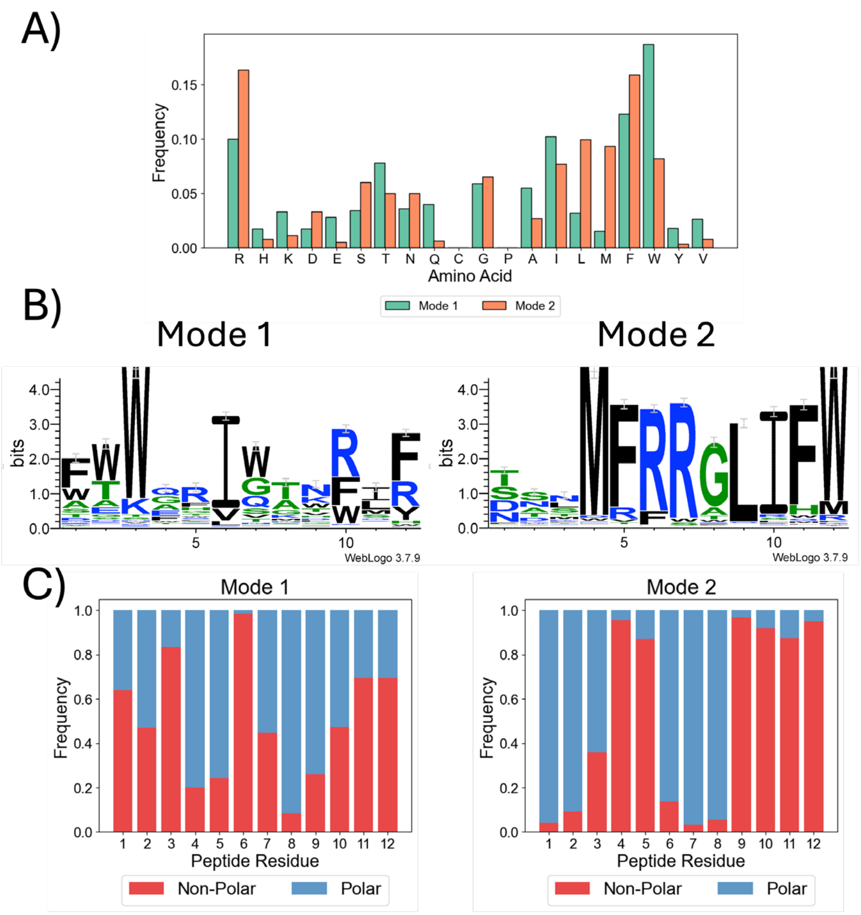

Figure S13. Difference in amino acid composition and patterning for the two modes of PP-Selective plastic-binding design. Refer to Fig. 3a and associated discussion for description of the two modes. (a) Amino acid composition. (b) Sequence logos generated by WebLogo3 for all peptides in either of the two modes. (c) Arrangement of non-polar and polar amino acids in the peptide sequence for the two modes.

Fig. S13 analyzes the amino acid composition of the two modes found by the LSTM-SA during the design of PP-selective peptides (see Fig. 3). Panel a compares the frequency of each amino acid of peptides in either mode. While some amino acids frequencies differ significantly between the two modes (primarily R and W), the frequencies are generally similar. Panel b compares logos[25] of the peptides, which includes information on where amino acids are positioned in the sequence. The logos show significant differences. Peptides in mode 2 tend to be very similar except for their N-terminus residues, while peptides in mode 1 are more heterogeneous. This is also reflected in panel c, which shows the arrangement of non-polar residues (ILE, LEU, MET, PHE,

TRP, TYR, and VAL) and polar residues (all other amino acids). Mode 2 shows a consistent organization of with a non-polar C-terminus, while Mode 1 has less consistency. The differences in the logos and residue patterning indicate that the peptides in Mode 1 differ significantly from those in Mode 2.

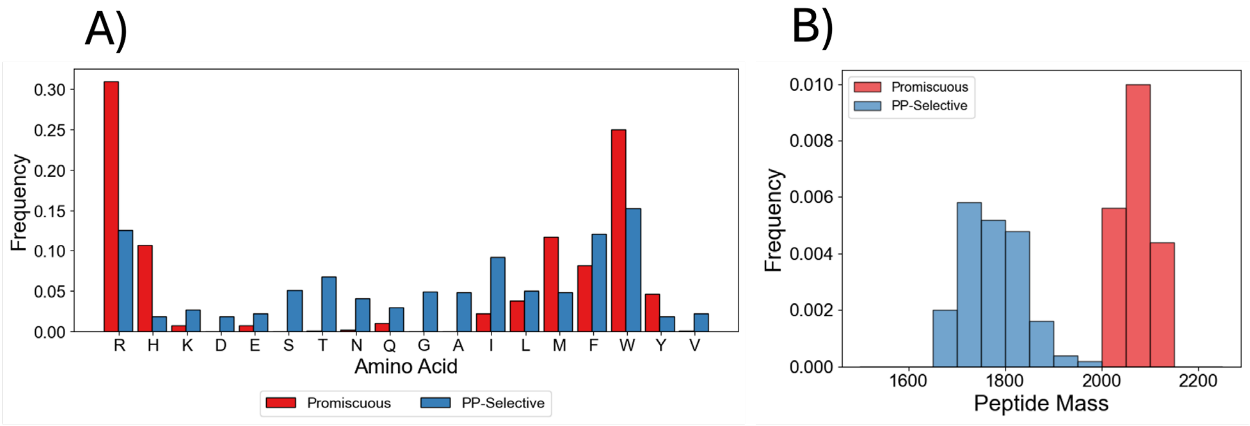

Figure S14. Comparison of physicochemical properties and amino acid composition of the promiscuous plastic-binding and PP-Selective peptides generated by the LSTM-SA. (a) Comparison of amino acid frequencies. (b) Comparison of peptide mass. Both panels analyze the 100 peptides with the best design score, either the highest average score for Promiscuous plastic-binding peptides, or the largest affinity score difference between PP and PET for PP-Selective peptides.

Fig. S14 analyzes the differences between the promiscuous plastic-binding and PP-selective peptides. We perform this comparison since the promiscuous plastic-binding peptides were found to also display PP-selectivity. Panel a compares the amino acid frequencies of the peptides, which differ significantly. A primary difference is the relative frequency of low mass amino acids, which are much more common in PP-selective peptides than promiscuous plastic-binding peptides. As shown in panel B, the result is that the PP-selective peptides tend to have a smaller molecular weight than promiscuous plastic-binding peptides.

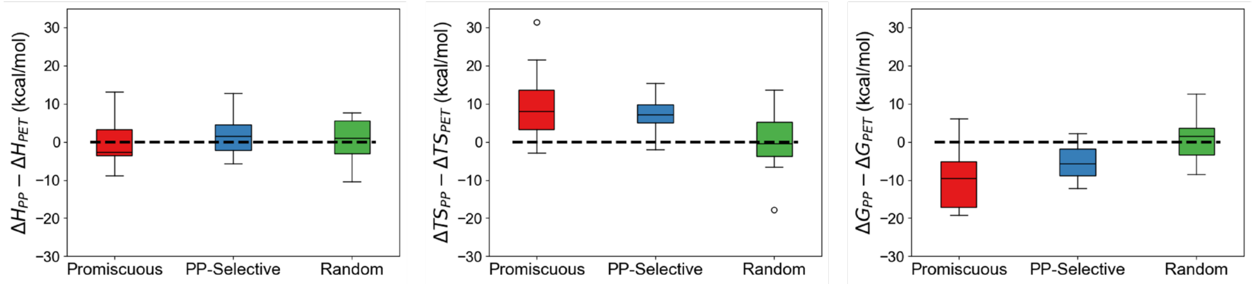

Figure S15. Comparison of MM/GBSA energy components of peptides when adsorbing to either polyethylene (PP) or PET. Three peptide categories are shown: Promiscuous plastic-binding, PP-Selective, and Random.

Fig. S15 compares the energy components from MM/GBSA calculations of peptides adsorbing to either polypropylene (PP) or PET. We analyze three peptide types: Promiscuous plastic-binding, PP-Selective, and Random. The data plotted are the distribution of the difference in energy for peptides between PP and PET. We perform this analysis to investigate why the PP-Selective and Promiscuous plastic-binding peptides are predicted to have greater affinity for polypropylene. As Random peptides show no consistent affinity difference, it provides a usual reference. For all three peptide types, van der Waals (VDW) energies are more favorable for PET, while the combination of electrostatic (ELE) and generalized born (GB) energies are more favorable for PP. The magnitude of the difference is roughly the same for all three peptide types. In contrast, the magnitude of the entropy difference (calculated using harmonic normal mode analysis) differs between the peptide types. There is a smaller entropic penalty for PP-selective and Promiscuous plastic-binding peptides adsorbing to PP than PET, while the entropic penalty is identical for the Random peptides adsorbing to the two plastics.

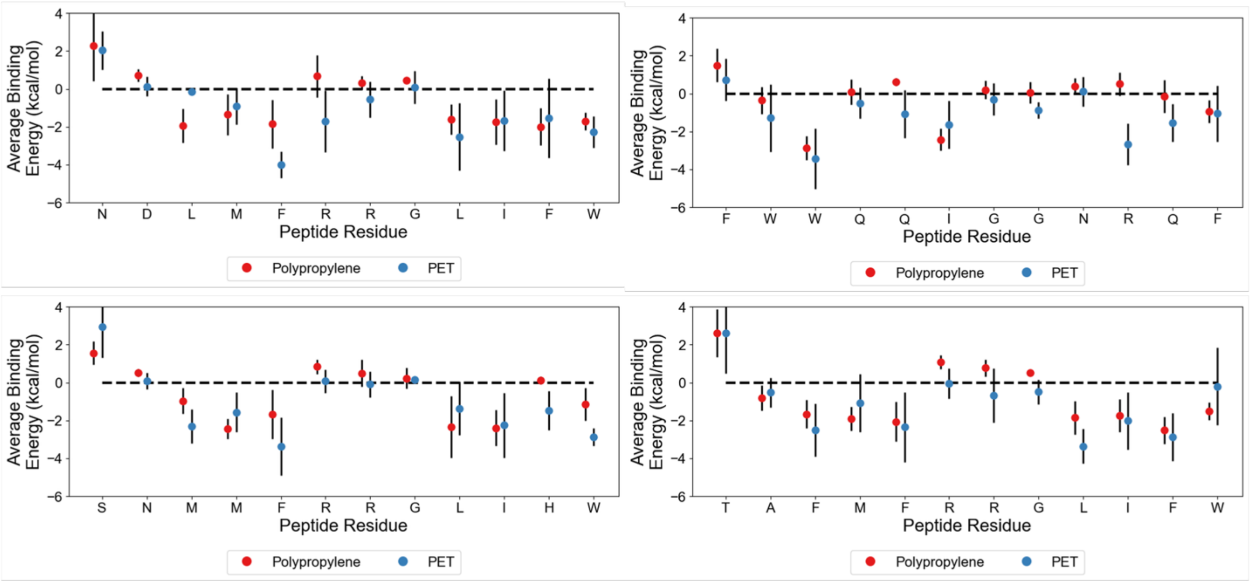

Figure S16. Per-residue energy decomposition of the most promising PP-selective peptides. The peptides analyzed are the four listed in Table 4. Red and blue circles indicate average interaction energy from the 8 simulations of each peptide adsorbing to polyethylene, polypropylene, respectively. Black bars indicate ± 1 standard deviation from the 8 simulations.

Fig. S16 compares the interaction energy for each residue for the four PP-selective peptides listed in Table 4. This analysis parallels that of the promiscuous peptides in Fig. S10.

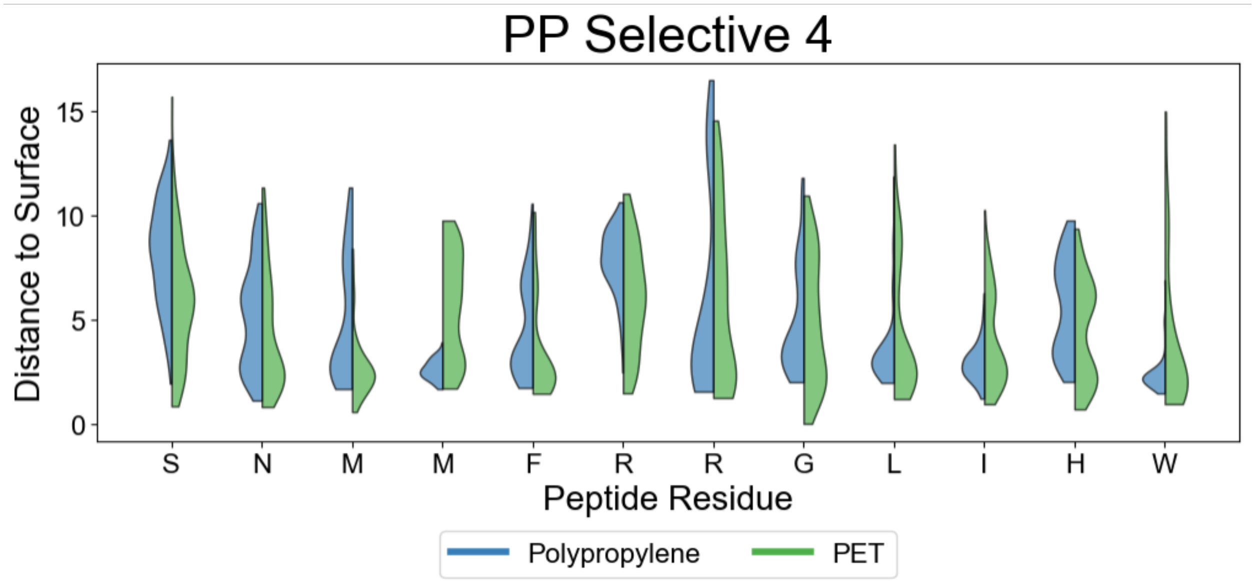

Figure S17. Per-residue distance to plastic surface of PP-selective Design 4 at the beginning of SMD simulations. Data is shown as violin plots, with results for polypropylene in blue and for PET in green.

Fig. S17 compares the adsorbed conformations of PP-selective Design 4 at beginning of SMD simulations. Comparing the distribution of distances of amino acids to the surface between the two plastics allows identification of amino acids with drastically different distributions. We hypothesize that amino acids with much denser distributions near the surface for PP than PET are responsible for PP selectivity, since proximity to the surface implies strong interactions with the plastic. For PP Selective 4, we find that M4, L9, I10, and W12 have more densely populated distributions near the PP surface than the PET surface, so these amino acids may give the overall PP-selectivity of the peptide.

Table S1. Model Architecture and trainable parameters of different recurrent networks used in this study.

Model	Architecture[1]	Trainable Parameters
**LSTM**	LSTM (2-layer, hidden dim=512, dropout=0.1) → Tanh → Linear	3191297
**BiLSTM**	BiLSTM (2-layer, hidden dim/2=256 per direction, dropout=0.1) → Tanh → Linear	2142721
**GRU**	GRU (2-layer, hidden dim=512, dropout=0.1) → Tanh → Linear	2393601
**RNN**	RNN (2-layer, hidden dim=512, dropout=0.1) → Tanh → Linear	798209
**Transformer**	Linear (18→512)→PositionalEncoding→TransformerEncoder (2-layer, hidden dim=512, nhead=4, ff_dim=2048, dropout=0.1) → Tanh → Linear	6315009

[1]A similar architecture is chosen across all models (2 layers with a hidden dimension of 512) to ensure a fair comparison.

A comparative analysis of different recurrent network architectures was conducted, including bidirectional LSTMs (BiLSTM), Gated Recurrent Units (GRUs), vanilla RNNs and Transformers was conducted. Table S4 summarizes the model architectures along with their respective trainable parameters. The BiLSTM model utilizes two bidirectional LSTM layers, each with a hidden dimension of 256 per direction, a Tanh activation, and a linear layer. The GRU and vanilla RNN based models maintain a similar architectural structure. The Transformer includes a Linear embedding layer on the one-hot encoded input features, to which the positional encoding is added and passed onto transformer encoder layers (as defined in PyTorch) followed by Tanh activation and a final linear layer.

Table S2. Statistics of promiscuous plastic-binding peptide affinity for plastics relative to Random peptides[1]

Description	PE	PP	PET	PVC	Nylon	Average
$\Delta G_{Promiscuous}$ - ΔG_{Random}	-9.9	-12.0	-1.9	-5.3	-2.3	-6.3

[1]Improvement defined as difference in binding energy between average ΔG (kcal/mol) of the LSTM-SA peptides for each plastic and the average ΔG of the Random peptides).

Table S2 compares the difference between the average adsorption free energy (ΔG) of promiscuous plastic-binding peptides and Random peptides. Results are provided for each of five plastics (PE, PP, PET, PVC, and nylon), as well as the average over all plastics. The more negative the value, the more affinity the promiscuous plastic-binding peptides have for that plastic relative to Random peptides. As all entries are negative, the promiscuous plastic-binding peptides on average outperform Random peptides. The affinity difference is large for PE and PP, intermediate for PVC, and small for PET and Nylon.

References

(1) Bergman, M. T.; Xiao, X.; Hall, C. K. In Silico Design and Analysis of Plastic-Binding Peptides. *J. Phys. Chem. B* **2023**, *127* (39), 8370–8381. https://doi.org/10.1021/acs.jpcb.3c04319.

(2) Wang, E.; Sun, H.; Wang, J.; Wang, Z.; Liu, H.; Zhang, J. Z. H.; Hou, T. End-Point Binding Free Energy Calculation with MM/PBSA and MM/GBSA: Strategies and Applications in Drug Design. *Chem. Rev.* **2019**, *119* (16), 9478–9508. https://doi.org/10.1021/acs.chemrev.9b00055.

(3) Vanquelef, E.; Simon, S.; Marquant, G.; Garcia, E.; Klimerak, G.; Delepine, J. C.; Cieplak, P.; Dupradeau, F.-Y. R.E.D. Server: A Web Service for Deriving RESP and ESP Charges and Building Force Field Libraries for New Molecules and Molecular Fragments. *Nucleic Acids Res.* **2011**, *39* (Web Server issue), W511-517. https://doi.org/10.1093/nar/gkr288.

(4) Vassetti, D.; Pagliai, M.; Procacci, P. Assessment of GAFF2 and OPLS-AA General Force Fields in Combination with the Water Models TIP3P, SPCE, and OPC3 for the Solvation Free Energy of Druglike Organic Molecules. *J. Chem. Theory Comput.* **2019**, *15* (3), 1983–1995. https://doi.org/10.1021/acs.jctc.8b01039.

(5) Hochreiter, S.; Schmidhuber, J. Long Short-Term Memory. *Neural Comput.* **1997**, *9* (8), 1735–1780. https://doi.org/10.1162/neco.1997.9.8.1735.

(6) Loshchilov, I.; Hutter, F. Decoupled Weight Decay Regularization. arXiv January 4, 2019. https://doi.org/10.48550/arXiv.1711.05101.

(7) Prechelt, L. Early Stopping - But When? In *Neural Networks: Tricks of the Trade*; Orr, G. B., Müller, K.-R., Eds.; Springer: Berlin, Heidelberg, 1998; pp 55–69. https://doi.org/10.1007/3-540-49430-8_3.

(8) Srivastava, N.; Hinton, G.; Krizhevsky, A.; Sutskever, I.; Salakhutdinov, R. Dropout: A Simple Way to Prevent Neural Networks from Overfitting. *J. Mach. Learn. Res.* **2014**, *15* (56), 1929–1958.

(9) Lundberg, S.; Lee, S.-I. A Unified Approach to Interpreting Model Predictions. arXiv November 25, 2017. https://doi.org/10.48550/arXiv.1705.07874.

(10) S. Alshehri, A.; T. Bergman, M.; You, F.; K. Hall, C. Biophysics-Guided Uncertainty-Aware Deep Learning Uncovers High-Affinity Plastic-Binding Peptides. *Digit. Discov.* **2025**, *4* (2), 561–571. https://doi.org/10.1039/D4DD00219A.

(11) Dhoriyani, J.; Bergman, M. T.; Hall, C. K.; You, F. Integrating Biophysical Modeling, Quantum Computing, and AI to Discover Plastic-Binding Peptides That Combat Microplastic Pollution. *PNAS Nexus* **2025**, *4* (2), pgae572. https://doi.org/10.1093/pnasnexus/pgae572.

(12) Vendrell, R. C.; Ajagekar, A.; Bergman, M. T.; Hall, C. K.; You, F. Designing Microplastic-Binding Peptides with a Variational Quantum Circuit–Based Hybrid Quantum-Classical Approach. *Sci. Adv. 10* (51), eadq8492. https://doi.org/10.1126/sciadv.adq8492.

(13) Case, D. A.; Aktulga, H. M.; Belfon, K.; Cerutti, D. S.; Cisneros, G. A.; Cruzeiro, V. W. D.; Forouzesh, N.; Giese, T. J.; Götz, A. W.; Gohlke, H.; Izadi, S.; Kasavajhala, K.; Kaymak, M. C.; King, E.; Kurtzman, T.; Lee, T.-S.; Li, P.; Liu, J.; Luchko, T.; Luo, R.; Manathunga, M.; Machado, M. R.; Nguyen, H. M.; O'Hearn, K. A.; Onufriev, A. V.; Pan, F.; Pantano, S.; Qi, R.; Rahnamoun, A.; Risheh, A.; Schott-Verdugo, S.; Shajan, A.; Swails, J.; Wang, J.; Wei, H.; Wu, X.; Wu, Y.; Zhang, S.; Zhao, S.; Zhu, Q.; Cheatham, T. E. I.; Roe, D. R.; Roitberg, A.; Simmerling, C.; York, D. M.; Nagan, M. C.; Merz, K. M. Jr. AmberTools. *J. Chem. Inf. Model.* **2023**, *63* (20), 6183–6191. https://doi.org/10.1021/acs.jcim.3c01153.

(14) Genheden, S.; Kuhn, O.; Mikulskis, P.; Hoffmann, D.; Ryde, U. The Normal-Mode Entropy in the MM/GBSA Method: Effect of System Truncation, Buffer Region, and Dielectric Constant. *J. Chem. Inf. Model.* **2012**, *52* (8), 2079–2088. https://doi.org/10.1021/ci3001919.

(15) Tribello, G. A.; Bonomi, M.; Branduardi, D.; Camilloni, C.; Bussi, G. PLUMED 2: New Feathers for an Old Bird. *Comput. Phys. Commun.* **2014**, *185* (2), 604–613.

(16) Jarzynski, C. Nonequilibrium Equality for Free Energy Differences. *Phys. Rev. Lett.* **1997**, *78* (14), 2690–2693.

(17) Case, D. A.; Cheatham III, T. E.; Darden, T.; Gohlke, H.; Luo, R.; Merz Jr., K. M.; Onufriev, A.; Simmerling, C.; Wang, B.; Woods, R. J. The Amber Biomolecular Simulation Programs. *J. Comput. Chem.* **2005**, *26* (16), 1668–1688. https://doi.org/10.1002/jcc.20290.

(18) Abraham, M. J.; Murtola, T.; Schulz, R.; Páll, S.; Smith, J. C.; Hess, B.; Lindahl, E. GROMACS: High Performance Molecular Simulations through Multi-Level Parallelism from Laptops to Supercomputers. *SoftwareX* **2015**, *1–2*, 19–25. https://doi.org/10.1016/j.softx.2015.06.001.

(19) Hess, B.; Bekker, H.; Berendsen, H. J. C.; Fraaije, J. G. E. M. LINCS: A Linear Constraint Solver for Molecular Simulations. *J. Comput. Chem.* **1997**, *18* (12), 1463–1472. https://doi.org/10.1002/(SICI)1096-987X(199709)18:12<1463::AID-JCC4>3.0.CO;2-H.

(20) Essmann, U.; Perera, L.; Berkowitz, M. L.; Darden, T.; Lee, H.; Pedersen, L. G. A Smooth Particle Mesh Ewald Method. *J. Chem. Phys.* **1995**, *103* (19), 8577–8593. https://doi.org/10.1063/1.470117.

(21) Bussi, G.; Donadio, D.; Parrinello, M. Canonical Sampling through Velocity Rescaling. *J. Chem. Phys.* **2007**, *126* (1), 014101. https://doi.org/10.1063/1.2408420.

(22) Berendsen, H. J. C.; Postma, J. P. M.; van Gunsteren, W. F.; DiNola, A.; Haak, J. R. Molecular Dynamics with Coupling to an External Bath. *J. Chem. Phys.* **1984**, *81* (8), 3684–3690. https://doi.org/10.1063/1.448118.

(23) Jorgensen, W. L. Quantum and Statistical Mechanical Studies of Liquids. 10. Transferable Intermolecular Potential Functions for Water, Alcohols, and Ethers. Application to Liquid Water. *J. Am. Chem. Soc.* **1981**, *103* (2), 335–340. https://doi.org/10.1021/ja00392a016.

(24) Maier, J. A.; Martinez, C.; Kasavajhala, K.; Wickstrom, L.; Hauser, K. E.; Simmerling, C. ff14SB: Improving the Accuracy of Protein Side Chain and Backbone Parameters from ff99SB. *J. Chem. Theory Comput.* **2015**, *11* (8), 3696–3713. https://doi.org/10.1021/acs.jctc.5b00255.

(25) Crooks, G. E.; Hon, G.; Chandonia, J.-M.; Brenner, S. E. WebLogo: A Sequence Logo Generator. *Genome Res.* **2004**, *14* (6), 1188–1190. https://doi.org/10.1101/gr.849004.
